# Low-Viscosity Route to High-Molecular-Weight Water-Soluble Polymers: Exploiting the Salt Sensitivity of Poly(*N*-acryloylmorpholine)

**DOI:** 10.1021/acs.macromol.3c02616

**Published:** 2024-02-23

**Authors:** Rory J. McBride, Elisa Geneste, Andi Xie, Anthony J. Ryan, John F. Miller, Adam Blanazs, Christine Rösch, Steven P. Armes

**Affiliations:** † Chemistry Department, University of Sheffield, Brook Hill, Sheffield S3 7HF, South Yorkshire, U.K.; ‡ Enlighten Scientific LLC, Hillsborough, North Carolina 27278, United States; § BASF SE, Carl-Bosch-Strasse 38, 67056 Ludwigshafen am Rhein, Germany

## Abstract

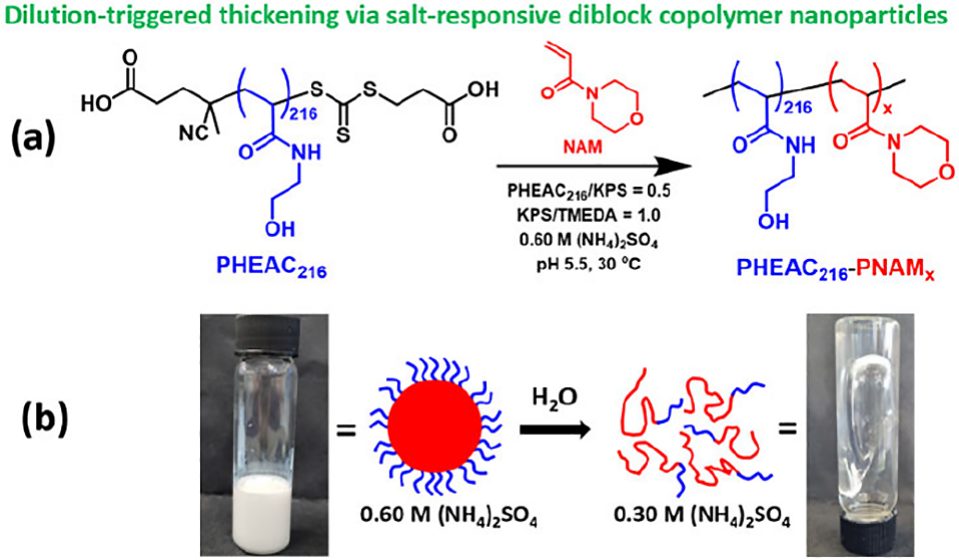

We report a new one-pot low-viscosity synthetic route to high molecular weight non-ionic water-soluble polymers based on polymerization-induced self-assembly (PISA). The RAFT aqueous dispersion polymerization of *N*-acryloylmorpholine (NAM) is conducted at 30 °C using a suitable redox initiator and a poly(2-hydroxyethyl acrylamide) (PHEAC) precursor in the presence of 0.60 M ammonium sulfate. This relatively low level of added electrolyte is sufficient to salt out the PNAM block, while steric stabilization is conferred by the relatively short salt-tolerant PHEAC block. A mean degree of polymerization (DP) of up to 6000 was targeted for the PNAM block, and high NAM conversions (>96%) were obtained in all cases. On dilution with deionized water, the as-synthesized sterically stabilized particles undergo dissociation to afford molecularly dissolved chains, as judged by dynamic light scattering and ^1^H NMR spectroscopy studies. DMF GPC analysis confirmed a high chain extension efficiency for the PHEAC precursor, but relatively broad molecular weight distributions were observed for the PHEAC–PNAM diblock copolymer chains (*M*_w_/*M*_n_ > 1.9). This has been observed for many other PISA formulations when targeting high core-forming block DPs and is tentatively attributed to chain transfer to polymer, which is well known for polyacrylamide-based polymers. In fact, relatively high dispersities are actually desirable if such copolymers are to be used as viscosity modifiers because solution viscosity correlates closely with *M*_w_. Static light scattering studies were also conducted, with a Zimm plot indicating an absolute *M*_w_ of approximately 2.5 × 10^6^ g mol^–1^ when targeting a PNAM DP of 6000. Finally, it is emphasized that targeting such high DPs leads to a sulfur content for this latter formulation of just 23 ppm, which minimizes the cost, color, and malodor associated with the organosulfur RAFT agent.

## Introduction

It is well known that water-soluble polymers can differ markedly in terms of their sensitivity toward added salt. For example, zwitterionic polymers such as poly(2-(methacryloyloxy)ethyl phosphorylcholine) are remarkably salt-tolerant and can remain water-soluble even in the presence of 5 M NaCl.^1^ On the other hand, the salt sensitivity of poly(2-(*N*-morpholino)ethyl methacrylate) (PMEMA) has been exploited to design several examples of “schizophrenic” AB diblock copolymers that can form either A-core or B-core micelles in aqueous media depending on the precise solution pH, temperature or salt concentration.^2−4^ In each case, PMEMA-core micelles were obtained upon addition of 0.7–1.0 M sodium sulfate.

Another well-known morpholine-functionalized water-soluble polymer is poly(*N*-acryloylmorpholine) (PNAM). The reversible addition–fragmentation chain transfer (RAFT) solution homopolymerization of NAM was first reported 20 years ago.^5−7^ More recently, PNAM has been used as the water-soluble steric stabilizer block for various polymerization-induced self-assembly (PISA) syntheses conducted in aqueous media.^8−11^ The non-ionic, highly biocompatible nature of PNAM has been exploited for various biomedical applications, including the sustained delivery of nitric oxide,^12^ as an alternative to PEGylation for protein conjugation,^13^ and the efficient harvesting of cell sheets from a micropatterned brush grown from a planar substrate.^14^ Given this prior literature, we were rather surprised to find that there are apparently no studies of the salt sensitivity of PNAM in aqueous solution, which is comparable to that observed for PMEMA.

The synthesis of various high molecular weight water-soluble polymers via RAFT solution polymerization has been explored by Destarac et al.,^15^ An and co-workers,^16−18^ and Sumerlin et al.^19,20^ Unfortunately, this approach inevitably leads to highly viscous solutions or gels, which makes further processing somewhat problematic. To address this problem, an inverse miniemulsion polymerization strategy has been recently developed by Olson et al.^21^ However, such formulations require use of an organic solvent (cyclohexane) and a relatively large amount of surfactant (Span 60) to stabilize the aqueous droplets.

Recently, we and others have developed RAFT aqueous dispersion polymerization formulations to prepare highly asymmetric double-hydrophilic diblock copolymers in the form of low-viscosity sterically stabilized particles.^22−24^ This is achieved by preparing a suitable salt-tolerant water-soluble polymer and then growing a salt-sensitive polymer from this precursor in the presence of sufficient added salt. For example, McBride et al. used a zwitterionic, cationic, or anionic precursor for the RAFT aqueous dispersion polymerization of *N*,*N*′-dimethylacrylamide (DMAC) in the presence of 2.0 M ammonium sulfate.^24^ Interestingly, DMAC conversions of more than 99% could be obtained at up to 20% w/w solids even when targeting PDMAC DPs as high as 5000. Similarly, Huang et al.^22^ statistically copolymerized acrylamide with 2-(methacryloyloxy)ethyl trimethylammonium chloride (METAC) and examined the resulting series of cationic water-soluble precursors for the RAFT aqueous dispersion polymerization of acrylamide in the presence of ammonium sulfate at 45–55 °C. METAC-rich copolymer precursors with higher DPs favored the formation of colloidally stable polyacrylamide-core particles. Narrow molecular weight distributions were achieved (typically *M*_w_/*M*_n_ = 1.10–1.20), but the target core-forming block DP was only varied from 600 to 1200, which is insufficient for optimal performance as an industrial flocculant.^25^ Moreover, the target solids content was relatively low at 3–6% w/w, which meant that the final acrylamide conversion was typically around 90–95%.^22^

In a related study, Bai et al.^26^ chain-extended a trithiocarbonate-capped poly(sodium 2-acrylamido-2-methylpropanesulfonate) (PAMPS) precursor via statistical copolymerization of sodium 2-acrylamido-2-methylpropanesulfonate (AMPS) with acrylamide in the presence of approximately 2.0 M ammonium sulfate. These RAFT aqueous dispersion polymerization syntheses were conducted under zero shear using a bifunctional precursor to produce ABA-type triblock copolymer particles of around 1–3 μm diameter at up to 20% w/w solids. Final comonomer conversions of up to 99% were achieved and GPC curves were obtained but no molecular weight data were reported.

**Scheme 1:**
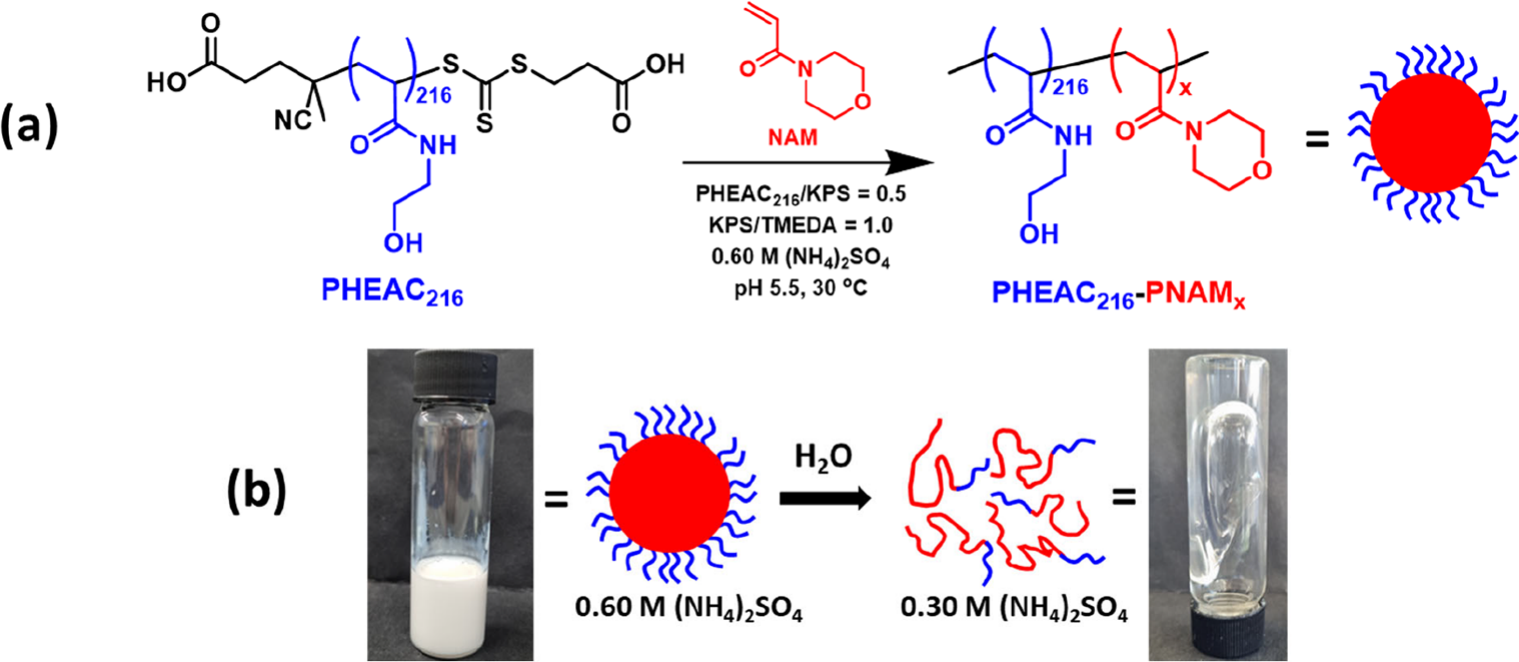
(a) Synthesis of **PHEAC**_**216**_**-PNAM***_**x**_* (*x* = 1000–6000) Diblock Copolymer Particles at 20% w/w Solids via RAFT Aqueous Dispersion Polymerization of NAM at 30 °C in the Presence of 0.60 M Ammonium Sulfate. Conditions: PHEAC_216_/KPS Molar Ratio = 0.50; KPS/TMEDA Molar Ratio = 1.0. (b) Schematic Cartoon and Corresponding Digital Images to Illustrate the Particle Dissolution that Occurs after Two-fold Dilution of the Initial Copolymer Dispersion with Deionized Water A two-fold dilution of this aqueous dispersion with deionized water halves the salt concentration and, hence, results in spontaneous dissociation of the particles, with the concomitant formation of a highly viscous transparent aqueous solution comprising molecularly dissolved diblock copolymer chains.

Herein we report the RAFT aqueous dispersion polymerization of NAM using a salt-tolerant water-soluble poly(2-hydroxyethyl acrylamide) (PHEAC) precursor in the presence of added salt (see Scheme 1). Unlike the prior studies described above, this formulation involves the synthesis of a wholly non-ionic diblock copolymer using a relatively low level of added salt (just 0.60 M ammonium sulfate). ^1^H NMR spectroscopy is used to study the kinetics of polymerization, molecular weight distributions are evaluated using DMF GPC, absolute weight-average molecular weights are determined by static light scattering in aqueous media, and the mean particle diameter is assessed using dynamic light scattering, laser diffraction and transmission electron microscopy. Finally, aqueous electrophoresis and rotational rheology are used to examine the particle surface charge and dispersion/solution viscosity, respectively.

## Experimental Section

### Materials

2-Hydroxyethyl acrylamide (HEAC, ≥96%), *N*-acryloylmorpholine (NAM, ≥97%), *N,N,N*′*,N*′-tetramethylethylenediamine (TMEDA, >99%) basic alumina, 1-butanethiol (>98.5%), 2-methyl-2-bromopropanoic acid (≥98%), carbon disulfide (anhydrous; 99%), 4-dimethylaminopyridine (DMAP; >99%), *N*,*N*′-dicyclohexylcarbodiimide (DCC; >99%), a 50% solution of 2-acrylamido-2-methyl-1-propanesulfonic acid sodium salt (AMPS), and deuterium oxide (D_2_O; ≥99.9% D) were purchased from Sigma-Aldrich Ltd. (U.K.). Potassium persulfate (KPS, 99%) and 2,2′-azobis(2-imidazolinylpropane) dihydrochloride (VA-044, ≥98%) were obtained from Fluorochem Ltd. (U.K.). Sodium hydroxide (≥98%) and ammonium sulfate (>98%) were sourced from Thermo Fisher Scientific (U.K.). 2-(Methacryloyloxy)ethyl phosphorylcholine (MPC) was kindly donated by Biocompatibles (U.K.). 2-(Acryloyloxy)ethyl trimethylammonium chloride (ATAC) was donated by BASF (Germany) in the form of an 80% w/w aqueous solution. PEO and PMMA standards were sourced from Agilent/PSS (Church Stretton, U.K.). The 4-(((2-(carboxyethyl)thio)carbonothioyl)thio)-4-cyanopentanoic acid RAFT agent (BM1433, >95%) used in this study was kindly donated by Boron Molecular (Victoria, Australia). *S*-Butyl-*S*′-(α,α′-dimethyl-α″-acetic acid)trithiocarbonate, methyl ester (MeBDMAT) was synthesized in-house. Each of the above chemicals was used as received. All solvents were purchased from Fisher Scientific (U.K.) and were used as received. Deionized water was used for all experiments.

### Synthesis Protocols

#### Synthesis of PHEAC Precursor via RAFT Aqueous Solution Polymerization of HEAC at 46 °C

HEAC (30.33 g, 0.26 mol), 4-((((2-carboxyethyl)thio)carbonothioyl)thio)-4-cyanopentanoic acid (BM1433; 0.270 g, 879 μmol), 2,2′-azobis(2-imidazolinylpropane) dihydrochloride (VA-044, 028.4 mg, 87.9 μmol), and deionized water (46.2 g) were weighed in a 250 mL round-bottom flask equipped with a magnetic flea, and the resulting reaction solution was degassed using a stream of nitrogen gas for 30 min at 20 °C. The sealed flask was immersed in an oil bath set at 46 °C, and the ensuing polymerization was allowed to proceed for 3 h to achieve 89% HEAC conversion. The aqueous polymer solution was purified by dialysis for 3 days to remove unreacted monomer and initiator and then freeze-dried overnight. The mean degree of polymerization was determined to be 216, as judged by end-group analysis using UV spectroscopy at the absorption maximum of 306 nm. DMF GPC analysis indicated an *M*_n_ of 23.9 kg mol^–1^ and an *M*_w_/*M*_n_ of 1.27.

#### Preparation of Aqueous Stock Solutions of 0.60 M Ammonium Sulfate, KPS Initiator, and TMEDA

Ammonium sulfate (39.65 g) was added to a 500 mL round-bottom flask, which was subsequently charged with deionized water to obtain a 0.60 M aqueous solution. Stock solutions of potassium persulfate (KPS; 5, 1 or 0.1% w/w) and *N,N,N*′*,N*′-tetramethylethylenediamine (TMEDA; 5, 1 or 0.1% w/w) were prepared using this 0.60 M (NH_4_)_2_SO_4_ aqueous solution. Each stock solution was degassed separately using a stream of nitrogen gas for 30 min at 20 °C.

#### Synthesis of PHEAC_216_–PNAM_*x*_ Diblock Copolymer Particles via RAFT Aqueous Dispersion Polymerization of *N*-Acryloylmorpholine (NAM) in 0.60 M Ammonium Sulfate at 30 °C

A typical protocol for the synthesis of PHEAC_216_–PNAM_3000_ diblock copolymer particles at 20% w/w solids was conducted as follows. The PHEAC_216_ precursor (40 mg, 1.59 μmol), NAM (675 mg, 4.78 mmol), and an aqueous solution of 0.60 M ammonium sulfate (1.628 g) were weighed into a 10 mL round-bottom flask charged with a magnetic flea and degassed with N_2_ for 30 min at 20 °C. This flask was then immersed in an oil bath set at 30 °C and KPS (3.19 μmol; 8.61 mg of a 0.1% w/w aqueous stock solution) and TMEDA (3.19 μmol; 370 mg of a 0.1% w/w aqueous stock solution) were added simultaneously to initiate the NAM polymerization. After 18 h, the final NAM conversion was judged to be more than 99% using ^1^H NMR spectroscopy (by comparing the integrated vinyl monomer signals at 5.7–6.6 ppm to the integrated acrylamide backbone signals at 2.5–2.8 ppm). DMF GPC analysis indicated an *M*_n_ of 117 kg mol^–1^ and an *M*_w_/*M*_n_ of 1.92. Higher PNAM DPs were targeted by reducing the concentration of the PHEAC_216_ precursor while maintaining a constant NAM concentration. Analogous methods were used for the synthesis of PATAC_242_–PNAM_3000_, PAMPS_230_-PNAM_3000_, and PMPC_139_–PNAM_3000_ nanoparticles for the “high salt” aqueous electrophoresis measurements.

#### Synthesis of MeBDMAT RAFT Chain Transfer Agent

The initial synthesis of *S*-butyl-*S*′-(α,α′-dimethyl-α″-acetic acid)trithiocarbonate (BDMAT) was based on protocols reported by Lai et al. and Bray et al.^27,28^

1-Butanethiol (24 mL), acetone (12 mL), and an aqueous solution of 5 M NaOH (44 mL) were added to a 500 mL round-bottom flask equipped with a magnetic flea and stirred for 25 min at 20 °C to produce a light pink solution. Upon addition of carbon disulfide (15 mL), the reaction solution turned orange, and stirring was continued for a further 30 min. Then, the flask was immersed in an ice bath. 2-Methyl-2-bromopropanoic acid (38.4 g) was heated to 50 °C (i.e., above its melting point range of 44–47 °C), and was slowly dripped into the ice-cold flask, which caused the reaction solution to turn yellow. The flask was removed from the ice bath, then the reaction mixture was poured into an aqueous solution of 5 M NaOH (44 mL), and the resulting solution was stirred for 20 h at 20 °C. This solution was diluted with deionized water (200 mL) and washed four times with *n*-hexane (4 × 200 mL). The orange aqueous phase was placed in a flask, which was immersed in an ice bath prior to the addition of an aqueous solution of 1.0 M HCl (230 mL) to produce a final solution pH of 3. The resulting yellow precipitate was isolated and washed with water prior to dissolution in chloroform (200 mL). After drying with anhydrous MgSO_4_ and removing the solvent under vacuum, the BDMAT product was isolated as a viscous orange-yellow liquid, which crystallized to form a yellow solid when poured into a glass vial. Subsequently, BDMAT (2.50 g, 9.92 mmol) and anhydrous dichloromethane (25.0 g) were added to an oven-dried 250 mL round-bottom flask equipped with a magnetic flea. This flask was immersed in an ice bath at 0 °C for 5 min. Then DMAP (279.0 mg, 2.28 mmol) and excess methanol (1.59 g, 49.6 mmol) were added and *N*,*N*′-dicyclohexylcarbodiimide (2.15 g, 10.4 mmol) was gradually added over 5 min. The reaction mixture was stirred overnight at 20 °C. The insoluble *N*,*N*′-dicyclohexylurea byproduct was removed via filtration, and the crude product was purified by silica column chromatography using dichloromethane as the mobile phase prior to drying in a vacuum oven overnight to isolate a viscous yellow oil; *S*-butyl-*S*′-(α,α′-dimethyl-α″-acetic acid)trithiocarbonate, methyl ester (MeBDMAT, 1.95 g, 74%), *m*/*z* 267 (M^+^), δ_H_ (400 MHz; CD_2_Cl_2_; (CH_3_)_4_Si) 0.97 (3 H, t, −CH_3_), 1.45 (2 H, q, −CH_2_−), 1.69 (8 H, s, −(CH_3_)_2_, q, −CH_**2**_−), 3.33 (2 H, t, −CH_2_−) 3.70 (3 H, s, −OCH_3_).

#### One-Pot Synthesis of PHEAC_220_-PNAM_6000_ Particles via RAFT Aqueous Dispersion Polymerization of NAM in the Presence of 0.60 M Ammonium Sulfate

The one-pot protocol for the synthesis of PHEAC_220_-PNAM_6000_ diblock copolymer particles at 20% w/w solids was conducted as follows. HEAC (952 mg, 8.27 mmol), MeBDMAT (10.0 mg, 37.6 μmol), and deionized water (315 mg; targeting 70% w/w solids) were weighed in a 250 mL round-bottom flask equipped with a magnetic flea, and the resulting reaction solution was degassed using a stream of nitrogen gas for 45 min at 20 °C. The flask was sealed using a rubber septum and immersed in an oil bath set at 30 °C. Then KPS (3.76 μmol; 101.6 mg of a 1.0% w/w aqueous stock solution) and TMEDA (3.76 μmol; 43.7 mg of a 1.0% w/w aqueous stock solution) were added to the reaction mixture via syringe. The ensuing HEAC polymerization was allowed to proceed for 3.5 h at 30 °C. Then a degassed mixture of NAM (15.2 g, 113 mmol) in an aqueous solution of 0.60 M ammonium sulfate (134.0 g) was added to target a final copolymer concentration of 20% w/w solids. Simultaneously, KPS initiator (75.2 μmol; 0.40 mL of a 5.0% w/w degassed aqueous stock solution) and TMEDA (75.2 μmol; 0.17 mL of a 5.0% w/w degassed aqueous stock solution) were also added to the reaction mixture. After stirring at 30 °C for 20 h, the final comonomer conversion was judged to be more than 99% using ^1^H NMR spectroscopy.

### Characterization Methods

#### ^1^H NMR Spectroscopy

Spectra were recorded in D_2_O at 25 °C using a 400 MHz Bruker Avance-400 spectrometer with 64 scans being averaged per spectrum.

#### UV Spectroscopy

Absorption spectra were recorded between 200 and 400 nm using a PC-controlled UV-1800 spectrophotometer at 20 °C and a 1 cm path length quartz cell. A series of four aqueous solutions of the 4-(((2-(carboxyethyl)thio)carbonothioyl)thio)-4-cyanopentanoic acid RAFT agent was used to construct a Beer–Lambert linear calibration curve (see Figure S1). The absorption maximum at 306 nm assigned to the π–π* transition for the trithiocarbonate group was used for this calibration plot, and the concentration range was selected such that the absorbance remained less than 1.0. The molar extinction coefficient was determined to be 9950 mol^–1^ dm^3^ cm^–1^, and the mean DP for the PHEAC precursor was calculated to be 216.

#### Gel Permeation Chromatography (GPC)

Molecular weights and dispersities were determined for the various homopolymers and diblock copolymers using an Agilent 1260 Infinity GPC instrument. This setup comprised a pump, a degasser, two PL-gel 5 μm Mixed-C columns in series, and a refractive index detector. HPLC-grade DMF containing 10 mM LiBr was used as the eluent, the column and detector temperature was set to 60 °C, and the flow rate was 1.0 mL min^–1^. Calibration was achieved using ten near-monodisperse poly(methyl methacrylate) standards (370–2,520,000 g mol^–1^), and data were analyzed using Agilent Technologies GPC/SEC software.

#### Static Light Scattering

A Dawn Helios II light scattering instrument (Wyatt Technology Corp.; equipped with a 130 mW linearly polarized gallium arsenide laser source operating at 658 mm and 18 detectors placed at angles ranging from 22.5 to 147°) was used to determine the absolute weight-average molecular weight (*M*_w_) of each diblock copolymer. This instrument was connected in series to an Agilent 1260 Infinity GPC instrument comprising a pump, a degasser, three GPC columns (PL-Aquagel Mixed-H, OH-30, and OH-40), and an Optilab T-rEX differential refractometer, which was used as a concentration detector in online mode. The eluent was an aqueous solution comprising 0.10 M NaNO_3_, 0.02 M TEA, and 0.05 M NaHCO_3_ at pH 8. The column and detector temperature was set to 30 °C, and the flow rate was 0.5 mL min^–1^. Copolymers were dissolved in the above GPC eluent at a relatively high concentration (>1 mg/mL) and injected using an autosampler. Data were analyzed using Astra 7 software according to the Zimm formalism. For Zimm plots, copolymer concentrations were varied by adjusting the injection volume. Thus 10, 25, or 50 μL of each copolymer solution was injected and a linear relationship was assumed between the injection volume and the light scattering detector signal.

#### Differential Refractive Index (d*n*/d*c*) Measurements

An Optilab T-rEX differential refractometer (Wyatt Technology Corp.) was used in batch mode to determine the d*n*/d*c* value for copolymers dissolved in the GPC eluent. Copolymer solutions of varying concentrations (0.5, 1.5, 2.5, 3.5, or 4.5 g dm^–3^) were injected consecutively (lowest concentration first) into the instrument at the same flow rate used for the online mode experiments using a syringe pump. A linear calibration plot of refractive index versus copolymer concentration enabled a d*n*/d*c* value to be calculated directly from the gradient. Hence the copolymer concentrations used for the online mode measurements can be calculated.

#### Transmission Electron Microscopy

Cu/Pd TEM grids (Agar Scientific, U.K.) were coated in-house with a thin film of amorphous carbon and then treated with a plasma glow discharge for 30 s to generate a hydrophilic surface. A 10 μL droplet of a freshly diluted 0.5% w/w aqueous copolymer dispersion was pipetted onto a hydrophilic grid for 1 min, then carefully blotted with filter paper to remove excess sample. Then a single 10 μL droplet of a 0.75% w/w aqueous solution of uranyl formate was pipetted onto the grid for 20 s to stain the deposited particles. Excess stain was carefully blotted and dried using a vacuum hose. Imaging was performed using an FEI Tecnai Spirit 2 microscope equipped with an Orius SC1000B camera and operating at an accelerating voltage of 80 kV.

#### Dynamic Light Scattering (DLS)

Analysis was performed using a Malvern Zetasizer Nano ZS instrument equipped with a 4 mW He–Ne 633 nm laser and an avalanche photodiode detector. The instrument was configured to automatically determine the experimental duration and optical attenuation. Each copolymer was diluted in 0.60 M (NH_4_)_2_SO_4_ to a concentration of 0.05% w/w and subsequently filtered through a 1.0 μm glass fiber filter. Backscattered light was detected at an angle of 173° and measurements were conducted at 20 °C using a 10 mm path length quartz cuvette cell. Malvern Zetasizer software v7.11 was used to calculate hydrodynamic diameters (*D*_h_) via the Stokes–Einstein equation, which assumes perfectly monodisperse, noninteracting spherical particles. Data were averaged over at least three consecutive runs with at least ten measurements being recorded for each run using the parameters listed in Table S1. Each dilute aqueous dispersion was passed through a 1 μm ultrafilter to remove dust prior to analysis. Standard deviations were calculated from the DLS polydispersity index (PDI) using the following relationship:
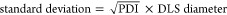


#### Laser Diffraction

Laser diffraction studies were performed on a Malvern Mastersizer 3000 instrument equipped with a Hydro EV dispersion unit and set at a stirring rate of 2000 rpm. A HeNe laser operating at 633 nm and a solid-state blue laser operating at 466 nm were used to analyze copolymer dispersions to measure the volume-average particle diameter. After each measurement, the cell was rinsed three times with water and the laser was aligned with the detector prior to data acquisition.

#### Rotational Rheology

An MCR 502 rheometer (Anton Paar, Graz, Austria) equipped with a concentric cylinder measuring set geometry (CC27) was used for rotational rheology experiments. Measurements were performed at 20 °C and shear sweeps were conducted from 0.05 to 500 s^–1^ with 51 measurements across the logarithmic shear rate ramp, with each measurement requiring 1–45 s (longer measurement times for lower shear rates). Approximately 10 mL of each copolymer dispersion (or solution) was used for each measurement. An overall measurement time of approximately 10 min was required for each sample. This precaution was necessary to ensure reliable data, particularly at lower shear rates.

#### Potentiometric Titration

Potentiometric titration was performed manually. 25.0 mL of acidified copolymer dispersion was placed in a 250 mL glass beaker and stirred with a magnetic flea. Titrant solution (0.6 M ammonium sulfate plus 0.2 M potassium hydroxide) was placed in a volumetric 50 mL buret, and a standard glass pH electrode was immersed in the aqueous dispersion. A total of 5.0 mL of titrant was added in aliquots of typically 0.5 mL, with smaller aliquots being used to determine the equivalence point of the titration. The apparent pH of the copolymer dispersion was recorded after addition of each aliquot and the solution pH re-stabilized within 30 s in each case. All pH measurements were performed at 22 ± 1 °C. Approximately 0.75 mL of the aqueous copolymer dispersion was removed at suitable intervals for subsequent electrophoretic light scattering (ELS) analysis. No attempt was made to remove dissolved CO_2_ or to prevent its dissolution. Thus it was assumed that these aqueous copolymer dispersions were saturated with dissolved CO_2_.

#### Electrophoretic Light Scattering

Electrophoretic mobilities were determined using NG-ELS (Next Generation Electrophoretic Light Scattering, Enlighten Scientific LLC, Hillsborough, NC). The functional design and operation of this instrument is based on the original phase analysis light scattering (PALS) apparatus,^29^ which employed a crossed-beam optical configuration (in contrast to the more common reference beam configuration used in commercial ELS instruments). The electrode assembly used for the NG-ELS equipment was based on that described by Uzgiris.^30^ Disposable polystyrene semi-micro cuvettes (4 mm path length) were used as the sample holders. Two identical parallel plate platinized platinum^31^ electrodes, 4 mm apart, were used to provide the driving voltage across the sample. The sample volume required for measurement was approximately 0.75 mL and aliquots of aqueous copolymer dispersions were analyzed without further dilution. A miniature NTC-type thermistor was placed in direct contact with each aqueous copolymer dispersion. This temperature probe was positioned at the midpoint between the electrodes and approximately 1 mm above the intersection point of the two laser beams. Temperature control was achieved by placing the sample cuvette in an aluminum block that ensured efficient heat transfer to a circulating water supply. The water temperature depended on the degree of Joule heating of the aqueous copolymer dispersion, which in turn depended on both its ionic conductivity and the applied voltage. Complex impedance analysis of the electrode waveform was used to quantify electrode polarization and Joule heating. Mobility measurements were made using sinusoidal electrode signal waveforms with an amplitude of 3.0 V at frequencies of either 32 or 64 Hz. The sample temperature was maintained at 24.5 ± 1.0 °C during measurement. The scattered light was analyzed for 60 s using both the PALS and the laser Doppler electrophoresis (LDE) methods simultaneously. i.e., the same data were used to calculate the electrophoretic mobility for each method. For each sample, ten independent measurements were made at each electrode signal frequency. This provided twenty measurements per sample from which a mean value and standard deviation were calculated.

## Results and Discussion

Over the past 25 years, various studies have demonstrated that PMEMA is a salt-intolerant water-soluble polymer.^2,3,32,33^ Herein we show that a second morpholine-functional water-soluble polymer, PNAM, exhibits similar behavior (see Table 1). More specifically, visual inspection studies (see digital photographs shown in Figure S2) confirm that PNAM_500_ homopolymer is soluble in the presence of 0.40 M ammonium sulfate at 30 °C but becomes insoluble when this salt concentration is increased to 0.60 M. Furthermore, NAM monomer remains water-miscible in the presence of 0.60, 1.0 or 2.0 M ammonium sulfate and only becomes water-immiscible at 3.0 M ammonium sulfate. In view of these observations, an ammonium sulfate concentration of 0.60 M was selected for the aqueous dispersion polymerization formulation explored in the present study. This salt concentration is significantly lower than that reported in the literature for a wide range of aqueous dispersion polymerization formulations conducted in the presence of ammonium sulfate.^22,23,40−42,24,26,34−39^

**Table 1:**
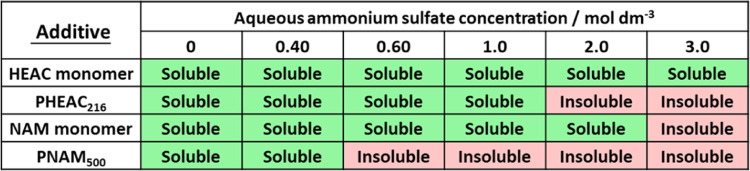
Summary of the Aqueous Solubilities of HEAC Monomer, NAM Monomer, PHEAC_216_ Homopolymer, and PNAM_500_ Homopolymer at 2.0% w/w Solids in the Presence of up to 3.0 M (NH_4_)_2_SO_4_ as Judged by Visual Inspection at pH 5.5 and 30 °C^a^

a Representative digital photographs were recorded above and below the critical salt concentration for each aqueous solution (see Figure S2).

Furthermore, we explore the first example of an aqueous dispersion polymerization formulation performed in the presence of salt in which solely non-ionic vinyl monomers are used to prepare both the relatively short steric stabilizer block and the much higher molecular weight core-forming block. More specifically, PHEAC is employed as the salt-tolerant steric stabilizer precursor and PNAM is selected as the salt-intolerant core-forming block (see Scheme 1). In striking contrast, all prior literature reports of similar aqueous dispersion polymerization syntheses conducted in the presence of salt involve using cationic, anionic, or zwitterionic comonomers.^22−24,26,34−39^ This is no doubt because these ionic components confer electrosteric stabilization. This mechanism ensures colloidal stability even in the presence of 2.0–3.0 M ammonium sulfate, i.e., an ionic strength 3–5 times higher than that employed in the current study.

**Figure 1:**
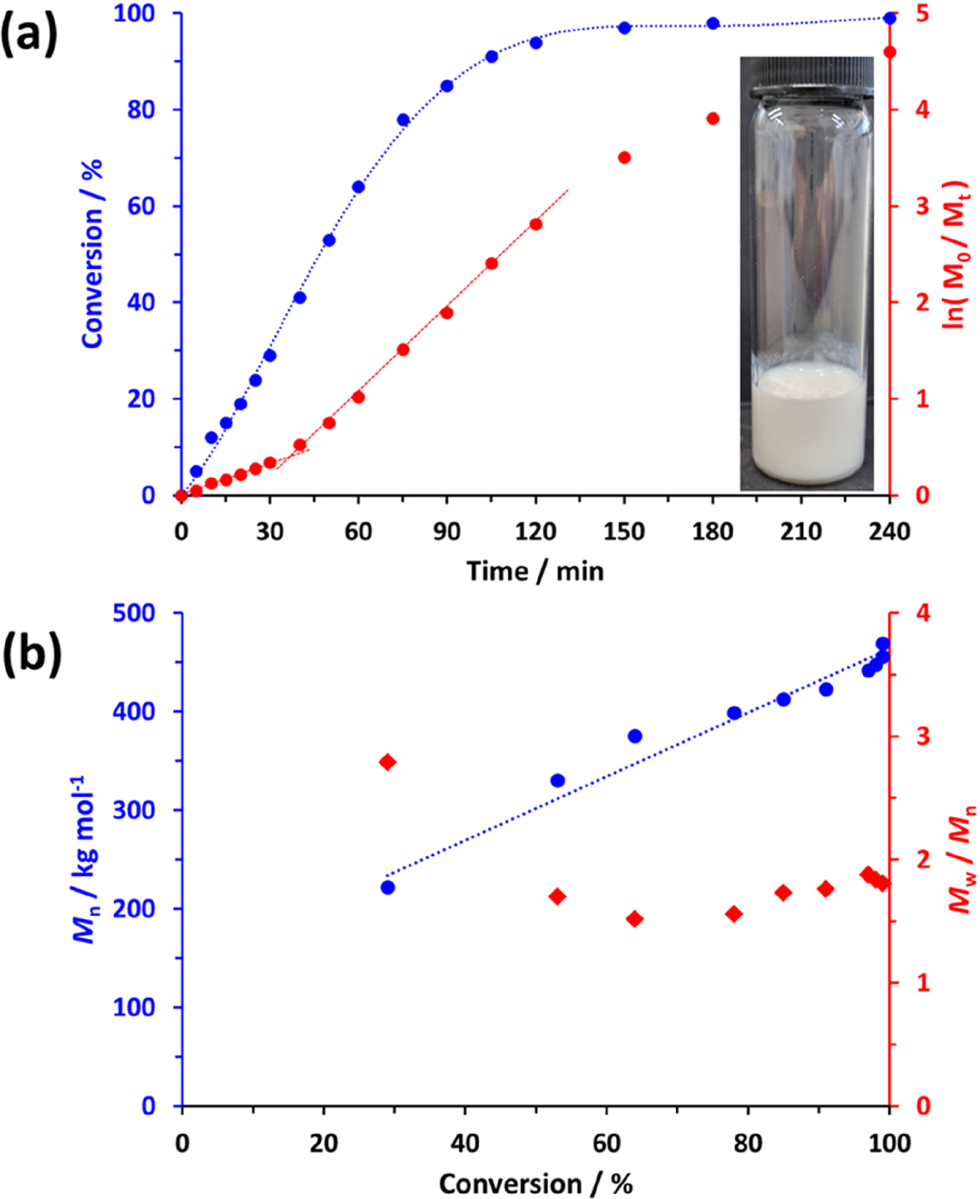
(a) Conversion vs time curve and corresponding semilogarithmic plot determined by ^1^H NMR spectroscopy for the RAFT aqueous dispersion polymerization of NAM at 30 °C in the presence of 0.60 M ammonium sulfate when targeting PHEAC_216_–PNAM_3000_ particles at 20% w/w solids. (b) Evolution of *M*_n_ and *M*_w_/*M*_n_ vs conversion as determined by DMF GPC analysis.

In an initial experiment, the RAFT aqueous dispersion polymerization of NAM is conducted at 30 °C in the presence of 0.60 M ammonium sulfate when targeting PHEAC_216_–PNAM_3000_ particles at 20% w/w solids. This reaction mixture is periodically sampled to enable the kinetics of the NAM polymerization to be monitored via ^1^H NMR spectroscopy. The resulting conversion vs time curve (blue data points) indicates that 95% conversion is achieved within 2 h and essentially full conversion is obtained within 3–4 h (see Figure 1). The corresponding semilogarithmic plot (red data points) reveals a change in gradient at around 38 min, which signifies the onset of particle nucleation.^43−46^ This occurs at approximately 42% NAM conversion, which corresponds to a mean PNAM DP of 1260. At this point, the unreacted NAM monomer diffuses into the nascent growing particles, which leads to a significantly faster rate of polymerization. The change in gradient indicates an approximately 2.6-fold increase in the rate of polymerization after nucleation. This is a more modest rate enhancement compared to that reported for other RAFT aqueous dispersion polymerization formulations.^43,47^ The final reaction mixture is a free-flowing, low-viscosity, turbid dispersion of PHEAC_216_–PNAM_3000_ particles (see inset photograph in Figure 1a). Selected aliquots taken during the above kinetic experiments are subjected to DMF GPC analysis (see Figure 1b). A linear evolution in *M*_n_ is observed with increasing conversion, which is characteristic of a RAFT polymerization. However, the *M*_w_/*M*_n_ values are relatively high at around 1.8. This is not unexpected given the relatively low PHEAC_216_/initiator molar ratio of 0.50 employed for these syntheses: this is sub-optimal for a well-controlled RAFT polymerization but essential to ensure a high final monomer conversion when targeting PNAM DPs of up to 7000.^22,26,48,,49^ In contrast, a BM1433/initiator molar ratio of 10 was employed for the synthesis of the PHEAC_216_ precursor, which had a relatively low dispersity (*M*_w_/*M*_n_ = 1.27).^50−52^ Subsequently, this precursor is employed to examine the effect of systematically varying the target DP from 1000 to 7000 for the salt-intolerant PNAM block (see Table 2).

**Table 2:** Summary of Monomer Conversions, DMF GPC Data, and Aqueous Static Light Scattering (SLS) Molecular Weight Data and Radii of Gyration for a Series of PHEAC_216_–PNAM_*x*_ Diblock Copolymer Particles (*x* = 1000–7000) Prepared by RAFT Aqueous Dispersion Polymerization of NAM in the Presence of 0.60 M Ammonium Sulfate (pH 5.5) Using a Redox Initiator at 30 °C for at least 18 h (See Scheme 1)

PNAM DP(*x*)	conversion/%	*M*_n_^a^/kg mol^–1^	GPC M_n_^b^/kg mol^–1^	*M*_w_/*M*_n_^b^	*M*_w_^c^ /kg mol^–1^	*R*_g_^c^/nm	c*^c^/mg mL^–1^	*D_z_*^d^/nm	PDI^d^
1000	>99	166	117	1.92	357			154	0.01
2000	98	302	166	2.35	548	20.2	110	228	0.03
3000	99	444	226	2.26	837	33.3	38	276	0.06
4000	99	584	401	2.24	1235	49.2	17	377	0.09
5000	96	702	538	2.29	1452	55.4	14	460	0.11
6000	97	846	766	2.11	2091	66.1	12	485	0.07
7000^e^	99	1002	855	2.21	macroscopic precipitation			570	0.09

a *M*_n_ corrected for the final NAM conversion, as determined by ^1^H NMR spectroscopy (assuming 100% chain extension efficiency).

b *M*_n_ and *M*_w_/*M*_n_ values, as determined by DMF GPC.

c The *M*_w_, radius of gyration (*R*_g_), and coil overlap concentration (c*) data are determined from aqueous SLS measurements.

d *D*_*z*_ denotes the *z*-average diameter and PDI denotes the polydispersity index, as determined by DLS studies.

e A macroscopic precipitate was obtained for this formulation, rather than a colloidally stable aqueous dispersion. For this reason, SLS analysis was not performed.

NAM conversions range from 96% to more than 99% for all seven aqueous PISA syntheses, indicating an efficient polymerization in each case. When targeting a PNAM DP of 1000, a low-turbidity dispersion of relatively high viscosity is obtained. In contrast, lower viscosity dispersions are obtained when targeting higher PNAM DPs. These observations are consistent with the kinetic data presented in Figure 1, which suggests that a minimum PNAM DP of 1260 is required for particle nucleation when targeting a PNAM DP of 3000. On the other hand, DLS studies of the PHEAC_216_–PNAM_1000_ formulation indicate a *z*-average diameter of approximately 154 nm (see later), which indicates that nucleation has already occurred when targeting this somewhat shorter PNAM block.

However, in view of its relatively high viscosity, this PHEAC_216_–PNAM_1000_ dispersion most likely contains a fraction of soluble copolymer chains in addition to sterically stabilized particles. This is understandable given that NAM monomer is a good solvent for PNAM. Clearly, the critical DP required for micellar nucleation depends on the target PNAM DP, no doubt because the latter parameter dictates how much unreacted NAM monomer is present at the onset of nucleation. Inspecting the penultimate column in Table 2, SLS studies (see Figure S3 for the corresponding Guiner plots) indicate a monotonic increase in the weight-average molecular weight (*M*_w_) for the PHEAC_216_–PNAM_1000–6000_ series. The same technique also enables the mean radius of gyration (*R*_g_) to be calculated for five of these six samples (the exception being for the lowest molecular weight copolymer, PHEAC_216_–PNAM_1000_). Finally, macroscopic precipitation is observed when targeting a PNAM DP of 7000, although a very high final monomer conversion is obtained even for this unsuccessful formulation. Thus a PNAM DP of 6000 appears to represent the effective upper limit for this new “low salt” RAFT aqueous dispersion polymerization, at least under the stated reaction conditions.

**Figure 2:**
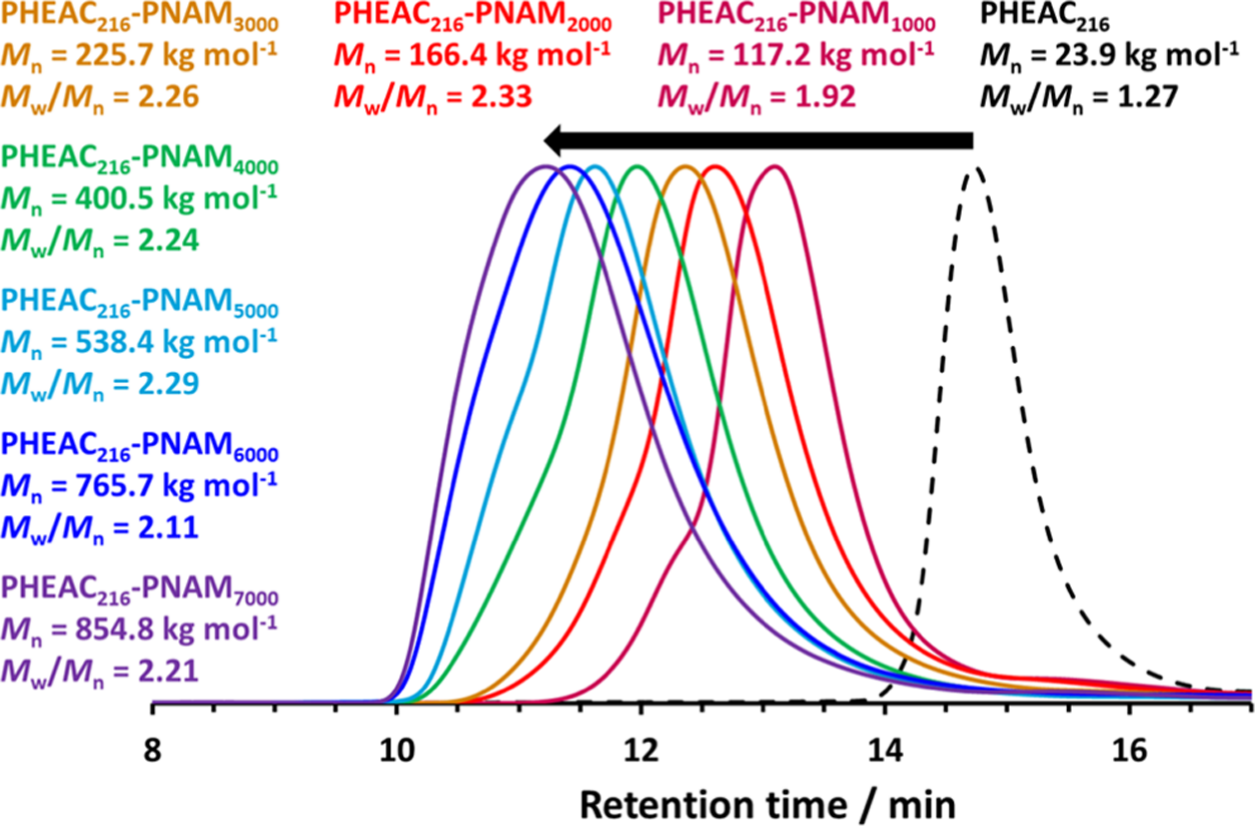
Normalized DMF GPC curves recorded for the PHEAC_216_ precursor and a series of PHEAC_216_–PNAM_*x*_ diblock copolymers prepared by its chain extension via RAFT aqueous dispersion polymerization of NAM at 30 °C in the presence of 0.60 M ammonium sulfate. *M*_n_ values are calculated relative to a series of near-monodisperse poly(methyl methacrylate) calibration standards.

One advantage of targeting non-ionic diblock copolymers is that such compositions are amenable to GPC analysis in common organic solvents. Indeed, the PHEAC–PNAM diblock copolymers investigated herein are soluble in DMF, which is a widely used GPC eluent. Accordingly, DMF GPC curves for a series of PHEAC_216_–PNAM_*x*_ diblock copolymers are shown in Figure 2. Increasing the target DP for the salt-intolerant PNAM block from 1000 to 6000 produces a monotonic increase in *M*_n_ while the dispersity (*M*_w_/*M*_n_) remains essentially constant. The discrepancy between the theoretical *M*_n_ values and the GPC *M*_n_ data simply reflects the use of poly(methyl methacrylate) calibration standards for GPC studies. Such standards inevitably incur a systematic error when used for the analysis of PHEAC_216_–PNAM_1000–6000_ diblock copolymers. Importantly, all six MWD curves are unimodal and efficient chain extension of the PHEAC_216_ precursor (see black curve) is achieved for each synthesis. This means that a well-defined diblock copolymer architecture is obtained in each case, although the dispersity of the PNAM block is undoubtedly high. This is not uncommon when targeting very high core-forming block DPs in PISA syntheses.^48,53,54^ For the present formulation, this is exacerbated by the relatively low [BM1433]/[KPS] molar ratio of 0.50 used for these aqueous PISA syntheses. As mentioned above, RAFT agent/initiator molar ratios of 5–10 are typically required for well-controlled RAFT polymerizations,^50−52^ although lower molar ratios of 0.5–3.3 are often utilized for PISA syntheses.^22,26,48,,49^ For the present system, a significantly lower molar ratio (i.e., a higher initiator concentration) was essential to ensure high final monomer conversions when targeting such high PNAM DPs. This pragmatic choice inevitably leads to inferior RAFT control, as evidenced by the relatively broad molecular weight distributions observed in Figure 2. However, such high dispersities can be beneficial if these salt-sensitive diblock copolymers are to be employed as viscosity modifiers (see later). One reviewer of this manuscript has suggested that our RAFT aqueous dispersion polymerization formulations are likely to generate PNAM homopolymer, as well as the target diblock copolymer chains. We cannot discount this possibility but we note that such contamination would have no adverse bearing on the intended application.

**Figure 3:**
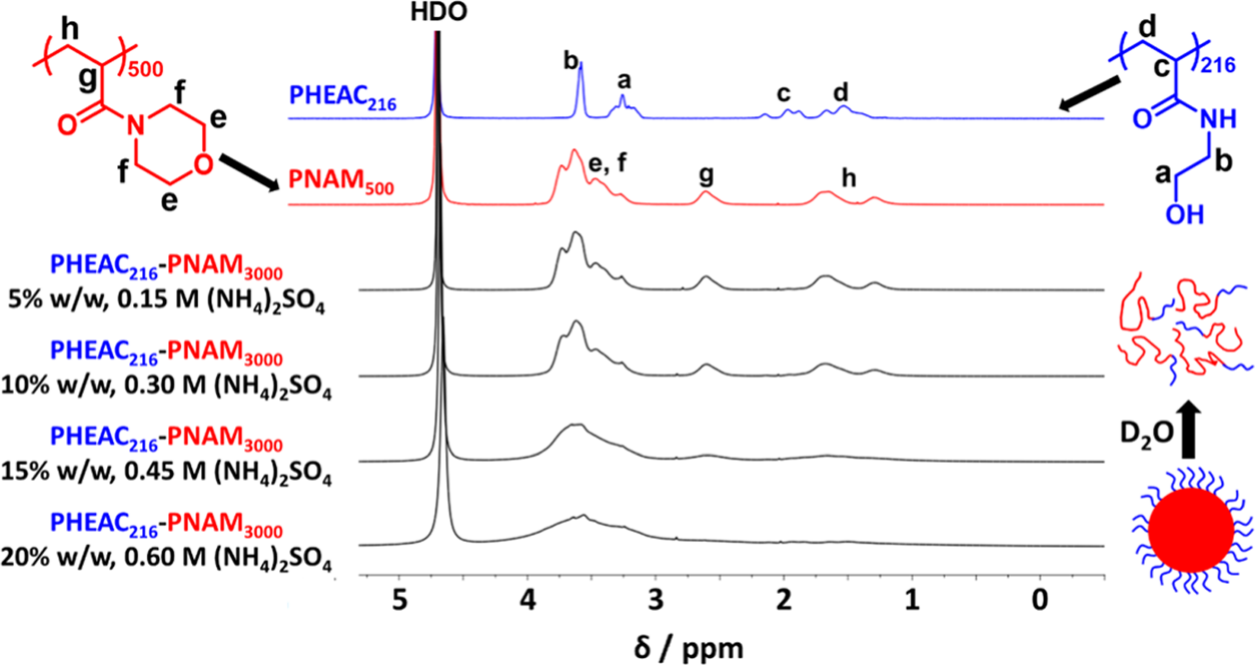
Partial ^1^H NMR spectra (D_2_O) recorded for a PNAM_500_ (red spectrum) homopolymer and a PHEAC_216_ (blue spectrum) homopolymer in the absence of salt, as well as PHEAC_216_–PNAM_3000_ diblock copolymer particles prepared in D_2_O at 20% w/w solids in the presence of 0.60 M ammonium sulfate, see lowest black spectrum. When this 20% w/w PHEAC_216_–PNAM_3000_ dispersion is diluted with D_2_O, both the background salt concentration and the corresponding copolymer concentration are systematically reduced (see other three black spectra).

A ^1^H NMR spectrum recorded for PHEAC_216_–PNAM_3000_ particles (see lowest black spectrum) prepared in D_2_O at 20% w/w solids in the presence of 0.60 M ammonium sulfate is shown in Figure 3. This sample is then serially diluted to 15, 10, and 5% w/w solids with D_2_O, and an NMR spectrum is recorded in each case. Visual inspection confirms that the initially turbid aqueous dispersion becomes completely transparent at either 10 or 5% w/w, indicating particle dissolution to afford molecularly dissolved copolymer chains. Notably, such transparent copolymer solutions are not colored, which is consistent with the relatively low level of RAFT agent required for such syntheses. To aid spectral assignments, NMR reference spectra are also recorded for a PNAM_500_ homopolymer (red spectrum) and a PHEAC_216_ homopolymer (blue spectrum) in the absence of salt. For the as-synthesized 20% w/w aqueous dispersion of PHEAC_216_–PNAM_3000_ particles, only a single broad signal at around 3.6 ppm is visible in the presence of 0.60 M ammonium sulfate, which is assigned to the relatively mobile pendent morpholine protons *e* and *f*. Under such conditions, the acrylamide backbone signals *g* and *h* are not detected. Nevertheless, this spectrum suggests partial (albeit low) hydration of the PNAM chains in the presence of 0.60 M ammonium sulfate. Dilution to 15% w/w using D_2_O lowers this salt concentration to 0.45 M. Under such conditions, the *e* and *f* signals are slightly better resolved. Moreover, the backbone proton signals *g* and *h* are now just about discernible at around 1.6 and 2.6 ppm. However, further dilution to 10% w/w is sufficient to cause particle dissociation because the salt-intolerant PNAM block becomes water-soluble in the presence of 0.30 M ammonium sulfate. Now the PNAM proton signals are essentially indistinguishable from those of the PNAM_500_ homopolymer dissolved in pure D_2_O (compare the uppermost black spectrum with the red spectrum). These observations are consistent with the schematic cartoon shown in Scheme 1b. It is also worth mentioning that there is no evidence for the PHEAC_216_ steric stabilizer in any of the four black spectra. However, this is understandable because this constitutes only a minor component of the overall diblock copolymer chains.

**Figure 4:**
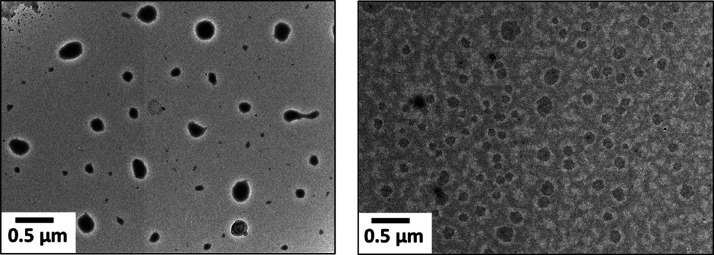
Representative TEM images recorded for PHEAC_216_–PNAM_6000_ particles.

TEM studies of the PHEAC_216_–PNAM_6000_ particles confirm the presence of spheres with a mean number-average diameter of 420 nm, see Figure 4. Given the highly asymmetric diblock composition, this suggests a kinetically trapped copolymer morphology. This is no doubt because the relatively long PHEAC DP confers effective steric stabilization, which is sufficient to prevent sphere–sphere fusion during the aqueous PISA synthesis.^55^

**Figure 5:**
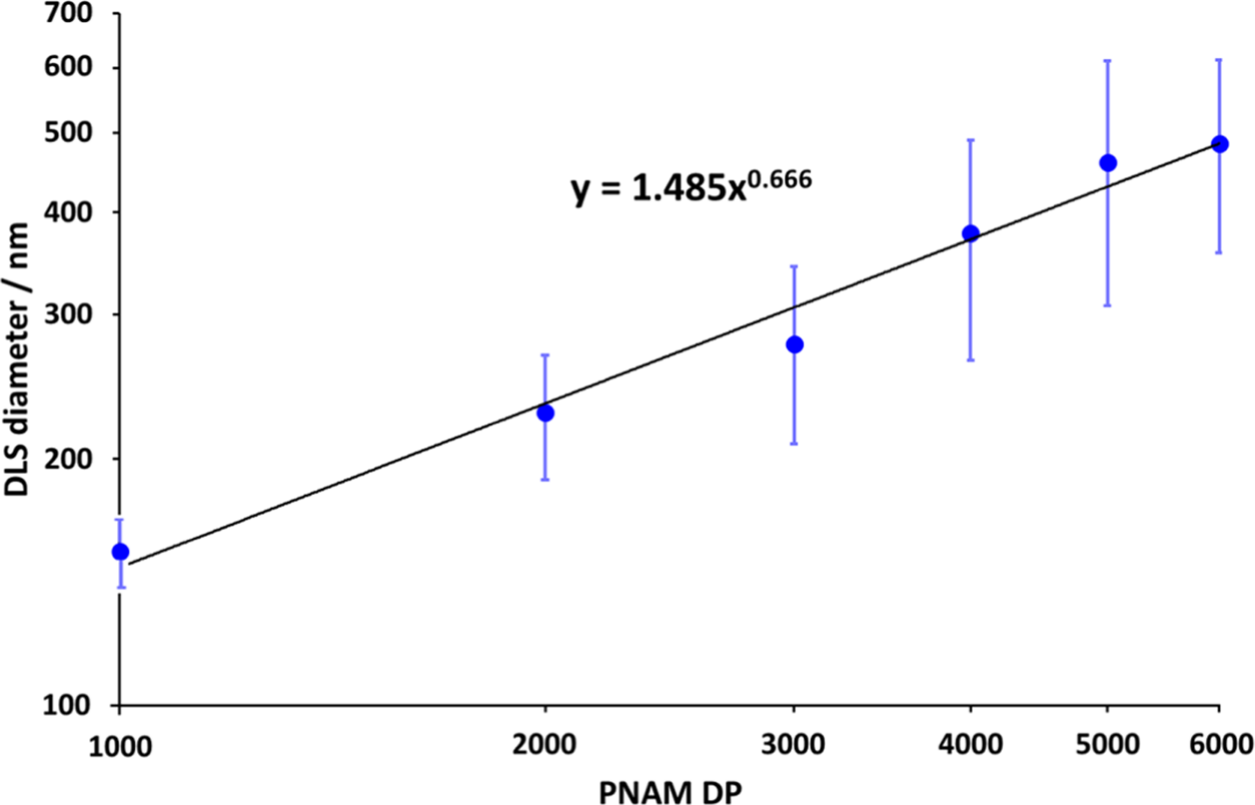
Relationship between the *z*-average diameter (reported by dynamic light scattering) and the mean degree of polymerization (DP) of the core-forming PNAM block for a series of six PHEAC_216_–PNAM_*x*_ particles (*x* = 1000–6000) prepared via RAFT aqueous dispersion polymerization of NAM at 30 °C in the presence of 0.60 M ammonium sulfate using a PHEAC_216_ precursor (see Table 1). [N.B. The standard deviations indicate the width of each particle size distribution rather than the experimental uncertainty].

DLS analysis of the six aqueous dispersions of PHEAC_216_–PNAM_1000–6000_ particles reveals relatively narrow particle size distributions with *z*-average diameters that increase monotonically with the PNAM DP. A log–log plot of this data set is shown in Figure 5.

If targeting a longer core-forming block leads to larger particles, there must be a corresponding increase in the inter-separation distance between neighboring steric stabilizer chains within the coronal layer. Eventually, this must result in ineffective steric stabilization, which accounts for the failure of the PHEAC_216_–PNAM_7000_ formulation (see Table 2). If the particles were fully dehydrated, the theoretical gradient for the linear plot shown in Figure 5 should be 0.50.^56−58^ However, this gradient is approximately 0.67, which suggests that the core-forming PNAM chains are partially hydrated in the presence of 0.60 M ammonium sulfate.^56−58^ This observation is consistent with the ^1^H NMR data discussed above. The six aqueous PHEAC_216_–PNAM_1000–6000_ dispersions are also analyzed using laser diffraction. This sizing technique reports comparable particle diameters to those obtained using DLS (see Figure S4).

Given that DMF GPC analysis only affords relative molecular weight data, static light scattering (SLS) is used to determine absolute *M*_w_ values for the series of six PHEAC_216_–PNAM_1000–6000_ copolymers. First, differential refractometry studies indicate a d*n*/d*c* value of 0.17 for such copolymers. For the SLS measurements, an online multi-angle laser light scattering detector (MALLS) is employed in combination with an aqueous GPC instrument. SLS experiments in aqueous solution are usually considered to be rather difficult owing to the ubiquitous presence of dust and/or air bubbles but fortunately the GPC columns act as an effective filtration system to minimize this problem.

**Figure 6:**
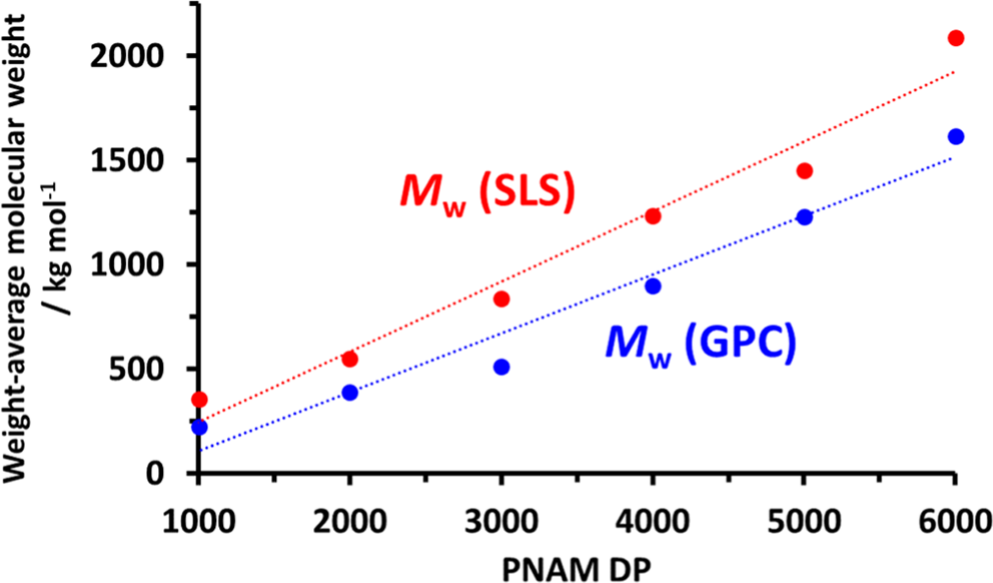
Relationship between the weight-average molecular weight (*M*_w_) and the mean degree of polymerization (DP) of the PNAM block for a series of six PHEAC_216_–PNAM_*x*_ diblock copolymers (*x* = 1000–6000) prepared via RAFT aqueous dispersion polymerization of NAM at 30 °C using a PHEAC_216_ precursor. The red data set was obtained by static light scattering studies, whereas the blue data set was calculated by multiplying each *M*_n_ value obtained by DMF GPC (see Table 2) by the corresponding dispersity (*M*_w_/*M*_n_).

Figure 6 shows the strikingly similar relationship between the absolute *M*_w_ data obtained from SLS studies and the apparent *M*_w_ values indicated by DMF GPC analysis (calculated by multiplying the *M*_n_ data shown in Table 2 by the corresponding dispersity). The difference between these two data sets is attributed to the systematic error incurred when using poly(methyl methacrylate) standards as GPC calibrants for the PHEAC_216_–PNAM_1000–6000_ chains.

**Figure 7:**
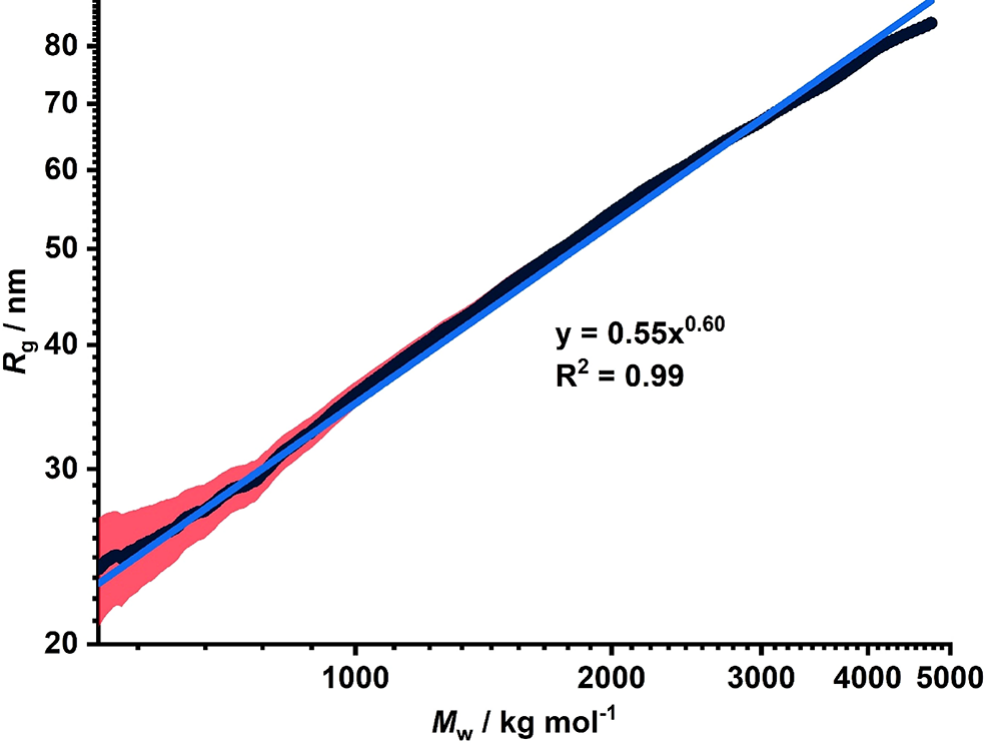
Relationship between the radius of gyration (*R*_g_) and the weight-average molecular weight (*M*_w_) obtained by SLS analysis of six PHEAC_216_−PNAM_1000-6000_ diblock copolymers via aqueous GPC using a MALLS detector. The MWDs of the six copolymers overlap (see black data) to provide a continuous relationship between *M*_w_ and *R*_g_. The blue line is a power law fit to these data: it has an exponent of 0.60, which indicates dilute copolymer coils in a good solvent. The uncertainty in *R*_g_ is greatest when the size of the polymer coils is much smaller than the wavelength of the incident light, as indicated by the red error bars in the low *M*_w_ regime.

The relationship between the radius of gyration, *R*_g_, and weight-average molecular weight, *M*_w_, is plotted in Figure 7. The global chain behavior follows a universal scaling law, *R*_g_ ∼ *M*^*v*^, where the Flory exponent, *v*, is related to the molecular weight, the solvent quality, and the inherent flexibility of the copolymer chain.^59^ Unperturbed flexible chains scale as *R*_g_ ∼ *M*_*w*_^1/2^, which is typical of a Gaussian conformation, In contrast, flexible chains in a good solvent follow self-avoiding walk statistics with *R*_g_ ∼ *M*_w_^3/5^, while relatively stiff chains behave like rigid rods, for which *R*_g_ ∼ *M*_w_.^60^

The behavior of semiflexible chains can be described using the worm-like chain (WLC) model, also known as the Kratky–Porod model.^61^ On sufficiently short length scales, semiflexible chains behave as rigid rods. However, over longer length scales, entropic considerations ensure that the copolymer chains form coils. For the WLC model, *R*_g_ can be expressed as a function of the contour length (molecular weight) and persistence length (intrinsic stiffness), according to the following relationship^61^
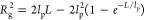
1where *L* is the contour length and *l*_p_ is the persistence length. A fit to the WLC model using the data set shown in Figure 7 gives *L* > *R*_g_ ≫ *l*_p_ ≈ 0.7 nm.

For each copolymer, we have calculated the coil overlap concentration (c*) as shown in eq 2, where *N*_A_ is Avogadro’s number^62^
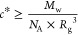
2The copolymer with the highest *M*_w_ (PHEAC_216_–PNAM_6000_) has c* = 12 g dm^–3^ (see Table 2). In a GPC measurement, the copolymer chains are fractionated according to their hydrodynamic volume and the most concentrated solutions for SLS analysis were 0.60 g dm^–3^ (PNAM DP = 2000) and 0.12 g dm^–3^ (PNAM DP = 6000). Hence, all SLS measurements were performed in the dilute solution regime. For a flexible copolymer chain in a good solvent, *R*_g_ ∼ *M*_w_^3/5^. According to Figure 7, the Flory exponent, *v*, is approximately 0.60,^62^ which indicates that the aqueous GPC eluent provides a good solvent environment for the PHEAC_216_–PNAM_*x*_ series.

**Figure 8:**
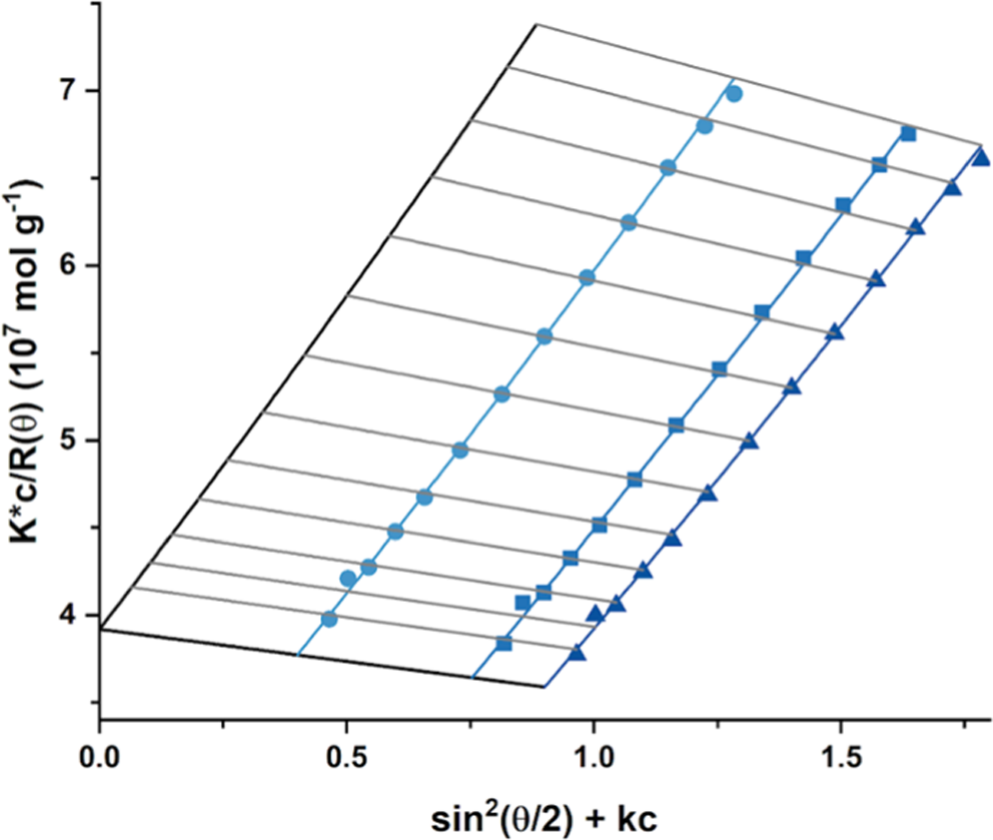
Zimm plot obtained by static light scattering analysis of PHEAC_216_–PNAM_6000_ diblock copolymer chains in aqueous solution (0.10 M NaNO_3_, 0.02 M TEA, and 0.05 M NaHCO_3_ at pH 8). The common intercept at 3.920 × 10^–7^ indicates an *M*_w_ of 2.5 × 10^6^ g mol^–1^.

A representative Zimm plot^63^ constructed for the highest molecular weight diblock copolymer (PHEAC_216_–PNAM_6000_) is shown in Figure 8. The double extrapolation to zero copolymer concentration and zero scattering angle yields a common intercept at 3.920 × 10^–7^, which corresponds to an *M*_w_ of 2.5 × 10^6^ g mol^–1^. This value is considered more accurate than the *M*_w_ of 2.09 × 10^6^ g mol^–1^ indicated in Table 2, which is calculated from the static light scattering plot shown in Figure S3. From the gradient for the zero-angle extrapolation, a second virial coefficient, *A*_2_, of −4.8 × 10^–5^ cm^3^ mol g^–1^ is calculated. Again, this suggests that the aqueous GPC eluent is a good solvent for the PNAM chains at 25 °C. This is consistent with our observation of inverse temperature solubility behavior for PNAM_500_ homopolymer, which remains soluble in deionized water up to 95 °C, as judged by turbidimetry studies (data not shown). Finally, the radius of gyration, *R*_g_, for five of the six diblock copolymers listed in Table 2 (PHEAC_216_–PNAM_2000–6000_) ranges from approximately 20 to 66 nm.

Two-fold dilution of each of the six turbid aqueous dispersions obtained from the successful PISA formulations summarized in Table 2 using deionized water produces a transparent viscous solution comprising molecularly dissolved copolymer chains. Rotational rheology studies of the latter aqueous solutions afford the viscosity vs shear rate data shown in Figure S5. Notably, each solution viscosity plateau observed at low shear (i.e., at shear rates of 0.05–5.0 s^–1^) correlates closely with the PNAM chain length.

**Figure 9:**
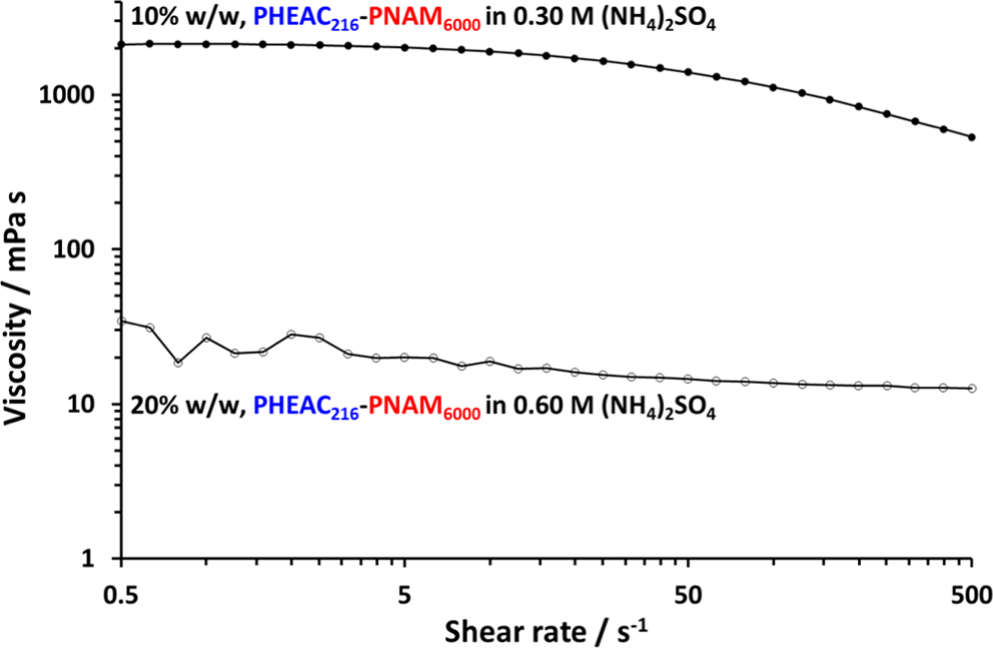
Viscosity vs shear rate plot obtained by rotational rheology studies of a 20% w/w aqueous *dispersion* of PHEAC_216_–PNAM_6000_ particles in the presence of 0.60 M (NH_4_)_2_SO_4_ compared to that for a 10% w/w aqueous *solution* of the same copolymer in the presence of 0.30 M ammonium sulfate (i.e., after a two-fold dilution using deionized water).

A viscosity vs shear rate plot recorded for the as-synthesized 20% w/w turbid aqueous dispersion comprising PHEAC_216_–PNAM_6000_ particles in the presence of 0.60 M ammonium sulfate confirms their Newtonian nature, see Figure 9.

A two-fold dilution of this dispersion with deionized water produces a transparent viscous aqueous solution (see Scheme 1) owing to molecular dissolution of the diblock copolymer chains in the presence of 0.30 M ammonium sulfate (see Figure 3). Each copolymer dispersion behaves as a Newtonian fluid and exhibits a viscosity that is almost independent of shear rate (see Figures 9 and S5). In contrast, the corresponding much more viscous copolymer solutions formed after two-fold dilution with water exhibit shear-thinning behavior at high shear. For the dispersions, the torque sensitivity of the rheometer is poor at low shear rates, which produces rather noisy data. The 10% PHEAC_216_–PNAM_6000_ copolymer solution exhibits a zero-shear plateau and shear-thinning behavior under applied shear (see Figure 9). Under zero-shear conditions, the solution viscosity is approximately two orders of magnitude higher for the final molecularly dissolved PHEAC_216_–PNAM_6000_ chains (≈2120 mPa s) compared to the initial aqueous dispersion of PHEAC_216_–PNAM_6000_ particles (≈20–35 mPa s). In principle, such dilution-triggered thickening could be useful for home and personal care product formulations. In this context, it is worth emphasizing that the relatively high copolymer dispersities are beneficial because the solution viscosity depends on the viscosity-average molecular weight, *M*_v_. Since this parameter correlates much more closely with *M*_w_ than *M*_n_, the approximately two-fold increase in *M*_w_ achieved by using a relatively high initiator concentration compared to that of the RAFT agent leads to a corresponding increase in the dilution-triggered thickening effect.

### One-Pot Synthesis of PHEAC_220_-PNAM_6000_ Diblock Copolymer Particles

**Scheme 2:**
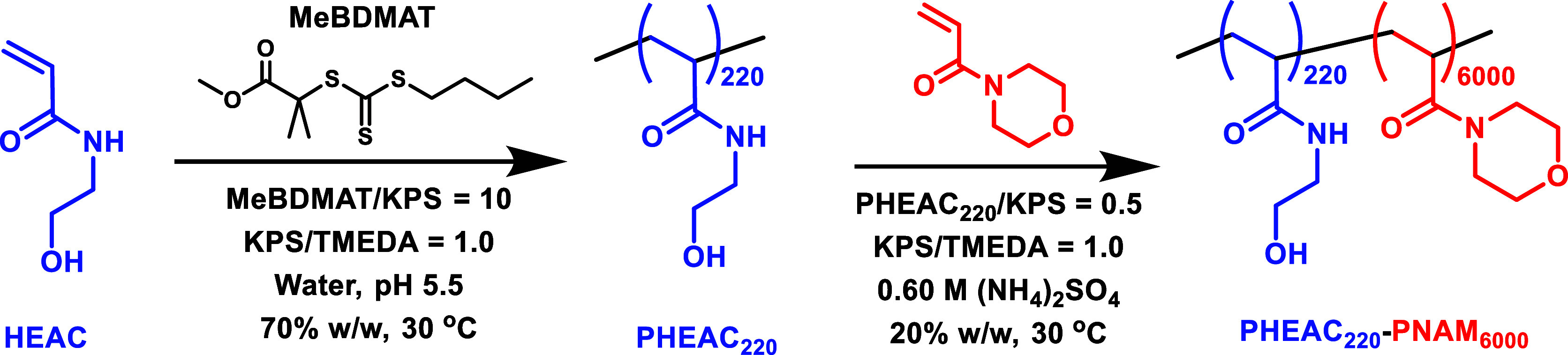
One-Pot Synthesis of Sterically Stabilized PHEAC_220_-PNAM_6000_ Diblock Copolymer Particles via (i) RAFT Aqueous Solution Polymerization of HEAC at 30 °C Targeting PHEAC_220_ at 70% w/w Solids Followed by (ii) RAFT Aqueous Dispersion Polymerization of NAM at 30 °C Targeting PHEAC_220_-PNAM_6000_ Particles at 20% w/w Solids

A water-soluble dicarboxylic acid-functionalized trithiocarbonate-based RAFT agent (BM1433) was selected for the aqueous PISA syntheses shown in Table 2. However, with the benefit of hindsight, this RAFT agent is mainly present in its anionic carboxylate form under the reaction conditions summarized in Scheme 1 (i.e., at pH 5.5). According to the aqueous PISA literature, such terminal anionic charge can enhance the colloidal stability of the final diblock copolymer nanoparticles via an electrosteric stabilization mechanism.^51,64,65^ Moreover, literature precedent suggests that a higher chain extension efficiency and a lower final dispersity are usually obtained when employing a one-pot synthesis protocol, rather than when utilizing a purified water-soluble precursor (e.g., PHEAC_216_ in the present study).^66−68^ Accordingly, we decided to explore the feasibility of conducting the one-pot synthesis of PHEAC_220_-PNAM_6000_ diblock copolymer particles using a non-ionic RAFT agent (MeBDMAT) under the reaction conditions shown in Scheme 2. The initial synthesis of the PHEAC_220_ precursor was conducted at 70% w/w solids to enable the HEAC monomer to act as a cosolvent for the otherwise water-insoluble MeBDMAT reagent.

Kinetic studies confirm that this first-stage polymerization proceeds to around 90% conversion within 3.5 h at 30 °C (see Figure S6). On subsequent addition of the NAM monomer, a turbid, free-flowing aqueous copolymer dispersion is obtained after a further 20 h at 30 °C, with visual inspection indicating no signs of macroscopic precipitation. ^1^H NMR spectroscopy studies confirm a final comonomer conversion of more than 99% and DLS analysis of the as-synthesized particles indicate a *z*-average diameter of 460 nm (DLS polydispersity = 0.06), which is comparable to the *z*-average diameter of 485 nm (DLS polydispersity = 0.07) reported for the PHEAC_216_–PNAM_6000_ particles prepared using the two-step protocol summarized in Scheme 1. This suggests that the terminal anionic carboxylate group on the PHEAC_216_ precursor has minimal effect on the size and colloidal stability of the final diblock copolymer particles. Moreover, this successful one-pot protocol augurs well for the potential scale-up of such “low salt” aqueous PISA formulations.

### Determination of Apparent ζ Potentials in the Presence of 0.60 M Ammonium Sulfate

**Scheme 3:**
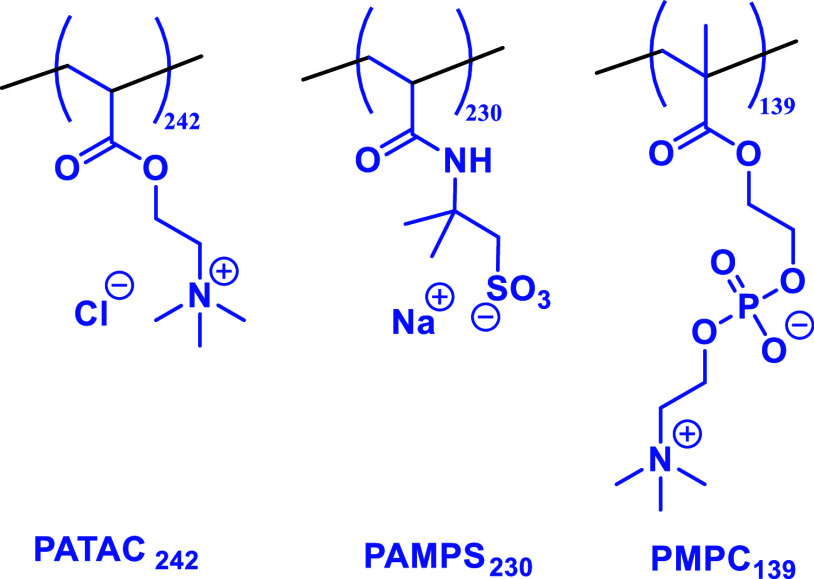
Chemical Structures of the Cationic PATAC_242_, Anionic PAMPS_230_, and Zwitterionic PMPC_139_ Precursors Used to Stabilize PNAM-Rich Diblock Copolymer Particles Prepared via RAFT Aqueous Dispersion Polymerization of NAM in 0.60 M (NH_4_)_2_SO_4_

Three alternative steric stabilizers were also evaluated for the RAFT aqueous dispersion polymerization of NAM conducted in the presence of 0.60 M ammonium sulfate. To complement the non-ionic salt-tolerant PHEAC_216_ steric stabilizer, we selected a cationic polyelectrolyte (PATAC_242_), an anionic polyelectrolyte (PAMPS_230_), and a zwitterionic polyelectrolyte (PMPC_139_), see Scheme 3. Each of these water-soluble polymers has been reported to exhibit salt-tolerant behavior.^22,24,26,35,38^ The target PNAM DP was 3000 and ^1^H NMR spectroscopy studies of the final reaction mixture confirmed that more than 99% NAM conversion is obtained in each case.

As discussed in our previous publications, aqueous electrophoresis experiments cannot be performed in highly salty media using conventional commercial instruments.^24,69^ Instead, we use a state-of-the-art instrument to determine the electrophoretic mobility for the PHEAC_216_–PNAM_3000_, PATAC_242_–PNAM_3000_, PAMPS_230_-PNAM_3000_ or PMPC_139_–PNAM_3000_ particles during the addition of known amounts of KOH in the presence of 0.60 M ammonium sulfate. The apparent pH of each aqueous copolymer dispersion was determined using an Ag/AgCl glass reference electrode without any compensation to offset the effect of the high ionic strength on the electrode response (although a temperature sensor within the electrode assembly did enable temperature compensation). Accordingly, the ζ potentials calculated using the Smoluchowski model^70^ are denoted as “apparent ζ potentials”.

**Figure 10:**
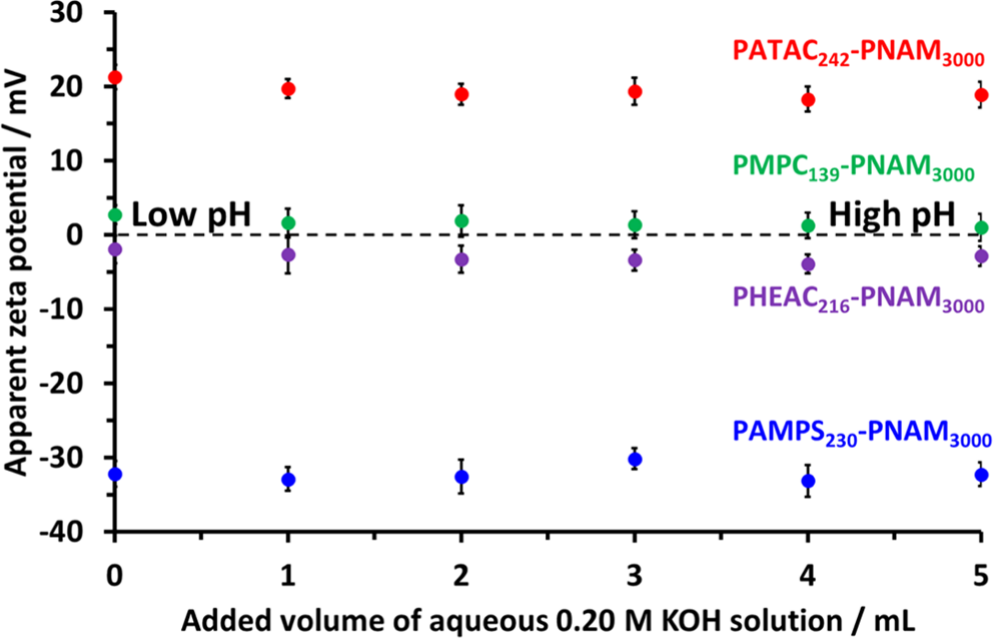
Apparent ζ potentials observed on gradual addition of 0.20 M KOH to 0.1% w/w aqueous copolymer dispersions containing 0.6 M ammonium sulfate: PHEAC_216_–PNAM_3000_ (purple circles), PATAC_242_–PNAM_3000_ (red circles), PAMPS_230_-PNAM_3000_ (blue circles), and PMPC_139_–PNAM_3000_ (green circles). Standard deviations are indicated for each data point.

Apparent ζ potential data determined by electrophoretic light scattering (ELS) for the four aqueous copolymer dispersions as a function of added KOH are shown in Figure 10. Data obtained for the PHEAC_216_–PNAM_3000_ particles prepared using the carboxylic acid–based RAFT agent are included, whereas the corresponding data obtained for PHEAC_216_–PNAM_3000_ particles prepared using a non-ionic RAFT agent are shown in Figure S7. Interestingly, there is no significant difference between these data sets. As expected, the characteristic electrophoretic footprint observed for each type of particle is dictated by the chemical nature of the steric stabilizer chains. Thus the cationic PATAC_242_–PNAM_3000_ particles possess positive apparent ζ potentials of +19.4 ± 1.6 mV, whereas the anionic PAMPS_230_-PNAM_3000_ particles exhibit negative apparent zeta potentials of −32.2 ± 1.8 mV. Finally, the zwitterionic PMPC_139_–PNAM_3000_ particles and the non-ionic PHEAC_216_–PNAM_3000_ particles exhibit near-zero apparent ζ potentials of +1.6 ± 1.8 and −3.0 ± 1.7 mV, respectively. These observations are consistent with previous data reported by us for such steric stabilizers.^24^

## Conclusions

The RAFT aqueous dispersion polymerization of NAM is conducted using a water-soluble salt-tolerant PHEAC precursor in the presence of a relatively low level of added salt (0.60 M ammonium sulfate) to produce a colloidal dispersion of sterically stabilized particles comprising wholly non-ionic diblock copolymer chains at 20% w/w solids.

Kinetic studies confirm that relatively high NAM conversions (94–99%) can be achieved within 2 h at 30 °C. DMF GPC studies indicate relatively efficient chain extension of the PHEAC precursor but relatively broad molecular weight distributions (*M*_w_/*M*_n_ > 1.92) for the final diblock copolymer chains. Systematic variation of the target DP for the salt-intolerant PNAM block leads to a progressive increase in the mean particle diameter as judged by dynamic light scattering and laser diffraction studies. Static light scattering (Zimm plot) studies indicate that *M*_w_ values of up to 2.5 × 10^6^ g mol^–1^ can be obtained using this aqueous PISA formulation.

^1^H NMR spectroscopy studies confirm that a two-fold dilution of an aqueous dispersion of such diblock copolymer particles using deionized water leads to their molecular dissolution. Rotational rheology studies indicate that the ensuing dilution-triggered thickening increases the solution viscosity by up to two orders of magnitude, which may be sufficient to offer potential commercial applications. Importantly, such high molecular weight copolymers contain minimal amounts of RAFT chain-ends: an as-synthesized 20% w/w aqueous dispersion of PHEAC_216_–PNAM_6000_ particles contains just 23 ppm sulfur, which is reduced to just 11.5 ppm sulfur for the final dilution-thickened aqueous solution.

## Supporting Information Available

UV absorption spectra and calibration plot; dynamic viscosities and refractive indices for various ammonium sulfate aqueous solutions; digital photographs recorded for salt solubility tests; six additional SLS plots; DLS and laser diffraction particle size distribution data; six viscosity vs shear rate plots obtained by rotational rheology studies of 10% w/w aqueous solutions of molecularly dissolved PHEAC_216_–PNAM_1000–6000_ chains in the presence of 0.30 M ammonium sulfate; conversion vs time curve for the synthesis of a PHEAC_220_ precursor; and additional ζ potential data (PDF)
