# Supplementary material for: Low-Viscosity Route to High-Molecular-Weight Water-Soluble Polymers: Exploiting the Salt Sensitivity of Poly(N-acryloylmorpholine)
Source: Macromolecules. 2024 Feb 23;57(5):2432–45. doi: 10.1021/acs.macromol.3c02616 (PMC10938879; doi:10.1021/acs.macromol.3c02616)
Supplement: Supplementary file 1 — ma3c02616_si_001.pdf [file ma3c02616_si_001.pdf]

Supporting Information for:

A Low-Viscosity Route to High Molecular Weight Water-Soluble Polymers: Exploiting the Salt Sensitivity of Poly(N-acryloylmorpholine)

R. J. McBride, E. Geneste, A. Xie, A. J. Ryan, A. Blanazs, C. Rösch, J. F. Miller and S. P. Armes*

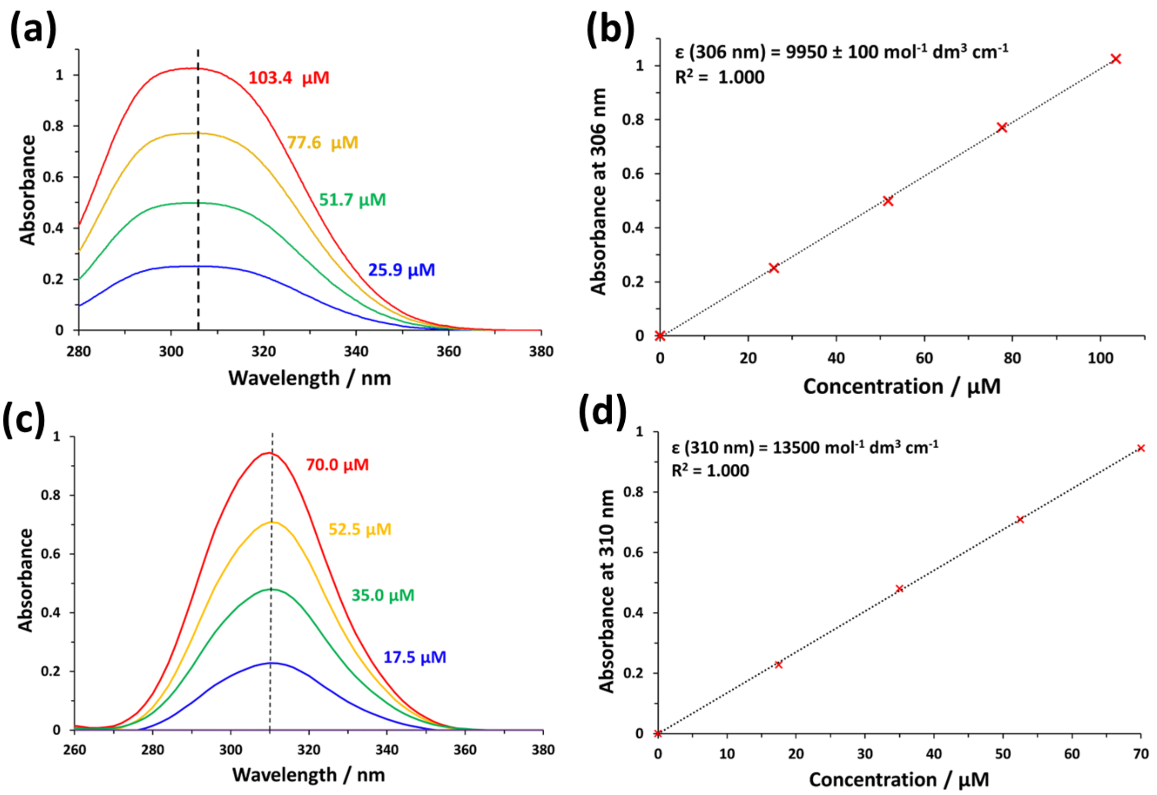

Figure S1. (a) UV absorption spectra recorded for 4-(((2-(carboxyethyl)thio)carbonothioyl) thio)-4-cyanopentanoic acid (BM1433) in water for a series of concentrations ranging from 0 µM to 103.4 µM. (b) Beer-Lambert calibration plot constructed for BM1433 in water to calculate its molar extinction coefficient (ε) at the absorption maximum of 306 nm. (c) UV absorption spectra recorded for S-butyl-S′-(α,α′-dimethyl-α′′-acetic acid)trithiocarbonate, methyl ester (MeBDMAT) in methanol for a series of concentrations ranging from 0 µM to 70.0 µM. (d) Beer-Lambert calibration plot constructed for MeBDMAT in methanol to calculate its molar extinction coefficient (ε) at the absorption maximum of 310 nm.

$(NH_4)_2SO_4$ concentration / M	Dynamic viscosity / Pa s	Refractive index
0.15	1.025	1.336
0.30	1.055	1.339
0.60	1.119	1.345
1.0	1.225	1.353
3.0	2.030	1.384

Table S1. Summary of dynamic viscosities and refractive indices for various ammonium sulfate aqueous solutions at 20 °C.[68] These data were used for DLS studies.

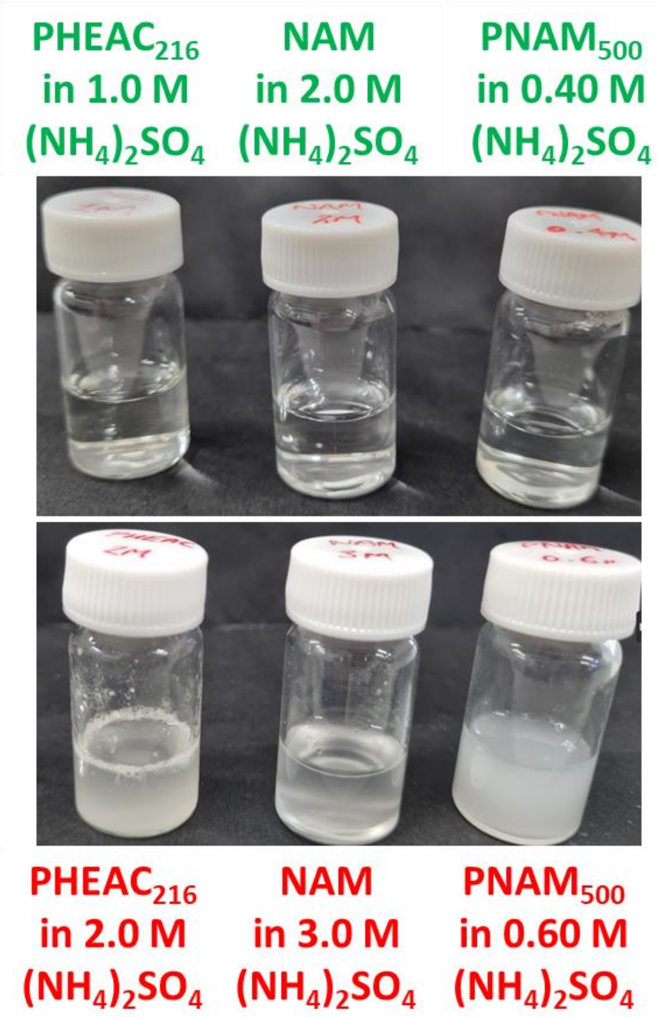

Figure S2. Aqueous salt solubility tests. Digital photographs recorded at 30 °C illustrating the visual appearance of 2.0% w/w aqueous solutions of NAM monomer, $PHEAC_{216}$ homopolymer and $PNAM_{500}$ homopolymer containing up to 3.0 M ammonium sulfate at pH 5.5.

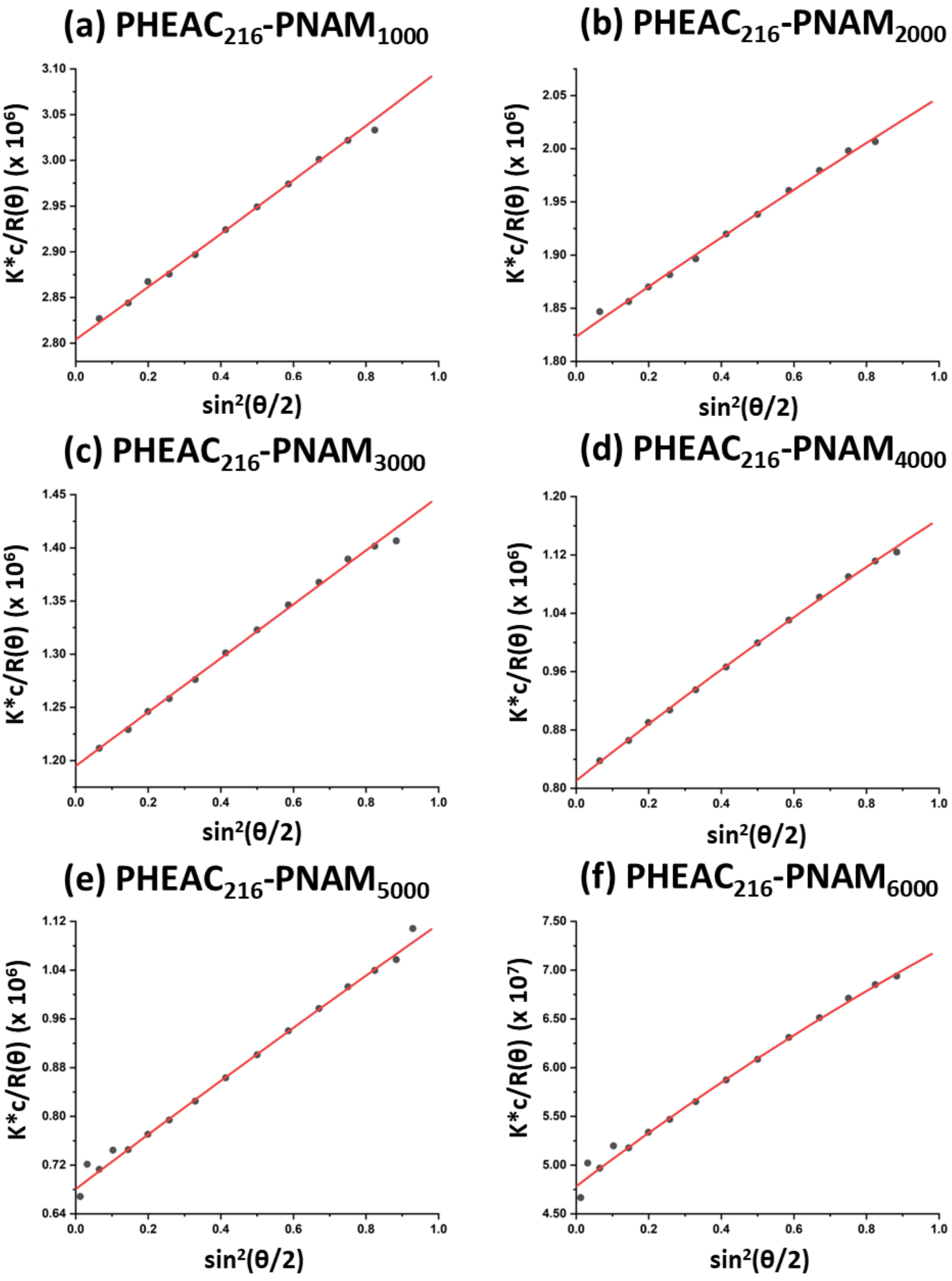

Figure S3. Static light scattering plots obtained using a MALLS-GPC detector to determine M_w values for six PHEAC$_{216}$-PNAM$_{1000\text{-}6000}$ diblock copolymers. Measurements were performed on dilute aqueous solutions in the presence of approximately 0.01 M ammonium sulfate.

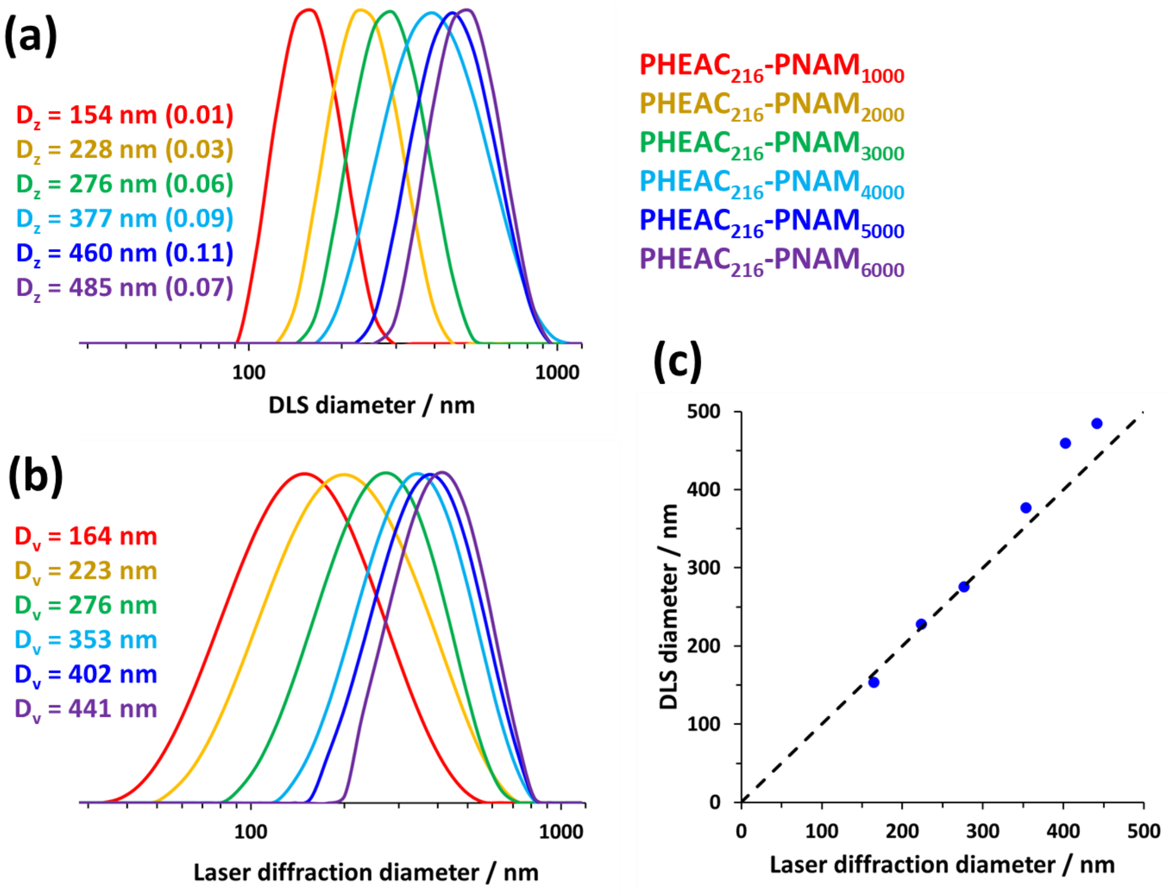

Figure S4. (a) DLS particle size distributions recorded for six aqueous dispersions of $PHEAC_{216}$-$PNAM_{1000\text{-}6000}$ particles (see **Table 2**). Z-average diameter and polydispersity data are indicated for each sample. (b) Laser diffraction particle size distributions recorded for the same six aqueous dispersions of $PHEAC_{216}$-$PNAM_{1000\text{-}6000}$ particles. Volume-average diameter data are indicated for each sample. (c) Comparison of DLS z-average diameter with laser diffraction volume-average diameter. A line of unity is included for reference.

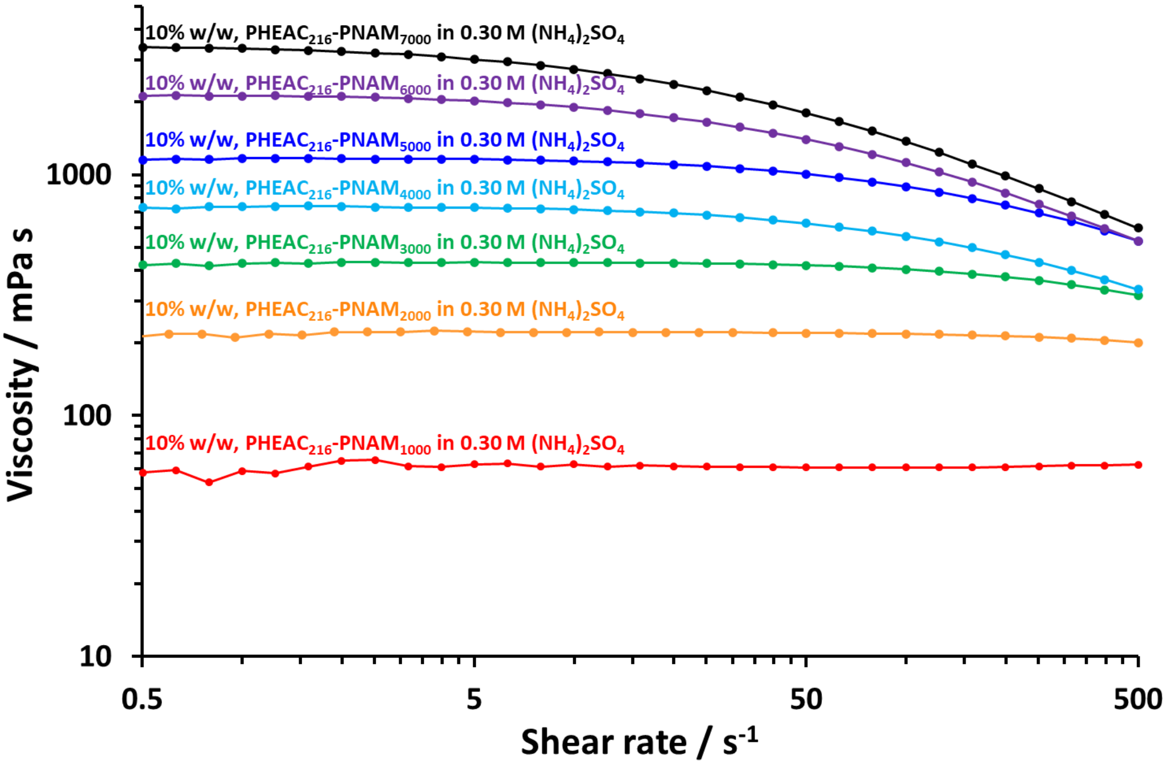

Figure S5. Viscosity vs. shear rate plots obtained by rotational rheology studies of 10% w/w aqueous *solutions* of molecularly-dissolved $PHEAC_{216}$-$PNAM_{1000-6000}$ chains in the presence of 0.30 M ammonium sulfate.

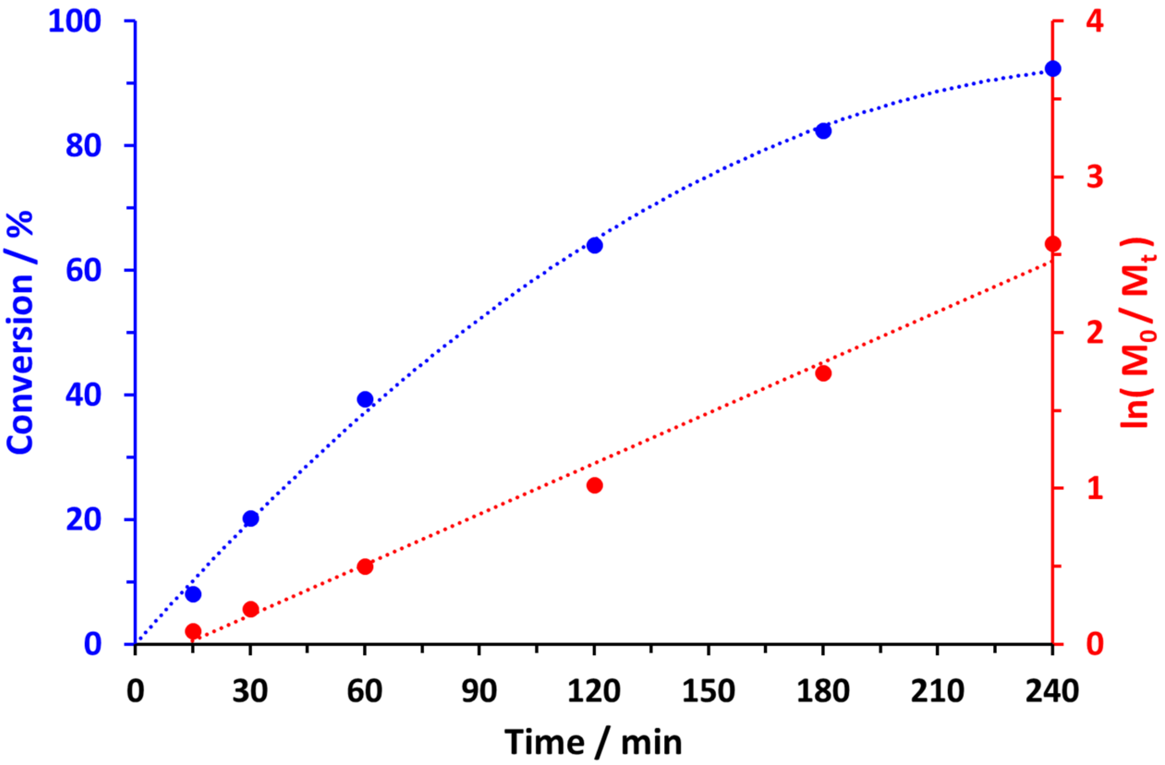

Figure S6. Conversion vs time curve obtained when targeting $PHEAC_{220}$ using MeBDMAT RAFT agent.

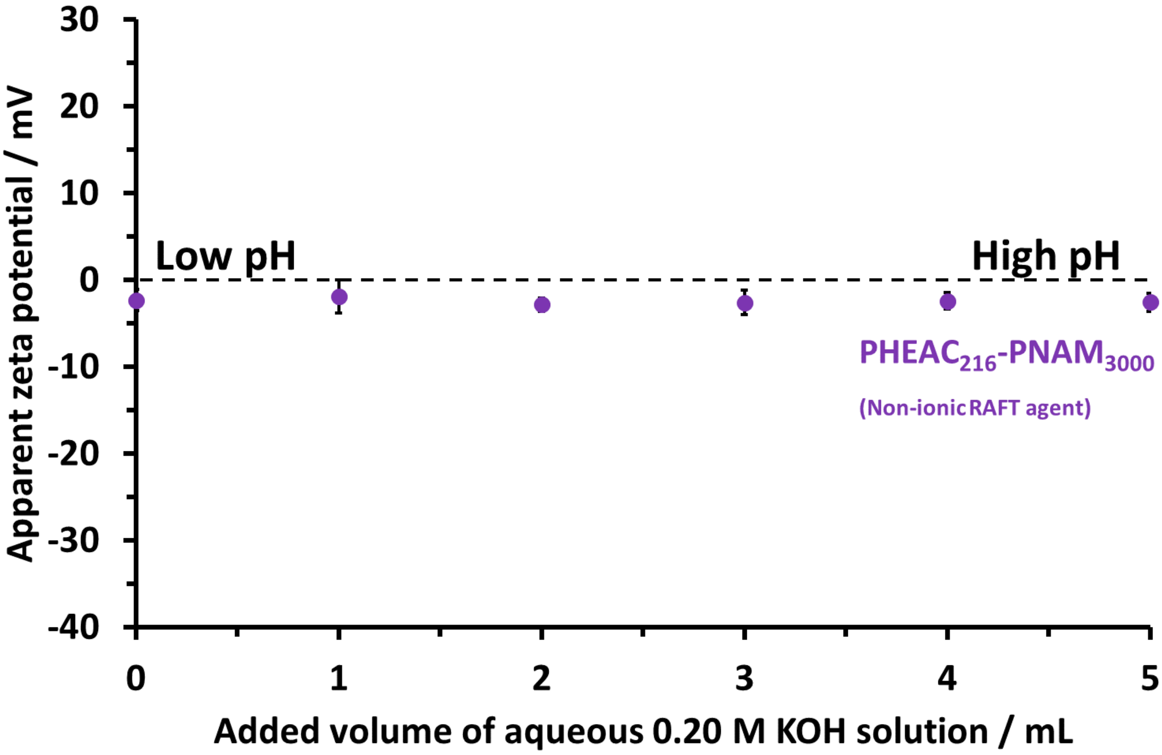

Figure S7. Apparent zeta potentials observed on addition of 0.20 M KOH to a 0.1% w/w aqueous dispersion of $PHEAC_{216}$-$PNAM_{3000}$ particles containing 0.6 M ammonium sulfate. Standard deviations are indicated for each data point.
